# Comparing Web-Based and Blended Training for Coping With Challenges of Flexible Work Designs: Randomized Controlled Trial

**DOI:** 10.2196/42510

**Published:** 2023-12-19

**Authors:** Sarah Elena Althammer, Anne Marit Wöhrmann, Alexandra Michel

**Affiliations:** 1 Federal Institute for Occupational Safety and Health Dortmund Germany; 2 Psychological Institute University Heidelberg Heidelberg Germany; 3 School of Management and Technology Leuphana University Lüneburg Lüneburg Germany

**Keywords:** blended training, web-based training, psychological detachment, well-being, work-life balance

## Abstract

**Background:**

Workers with flexible work designs (FWDs) face specific challenges, such as difficulties in detaching from work, setting boundaries between work and private life, and recovering from work.

**Objective:**

This study evaluated the effectiveness of an intervention in improving the recovery, work-life balance, and well-being of workers with FWDs compared with a waitlist control group. It also compares the effectiveness of a web-based training format and blended training format.

**Methods:**

In the web-based training format, participants individually completed 6 web-based modules and daily tasks over 6 weeks, learning self-regulation strategies to meet the particular challenges of FWDs. In the blended training format, participants attended 3 group sessions in addition to completing the 6 web-based modules. In a randomized controlled trial, participants were assigned to a web-based intervention group (196/575, 34.1%), blended intervention group (198/575, 34.4%), or waitlist control group (181/575, 31.5%). Study participants self-assessed their levels of primary outcomes (psychological detachment, satisfaction with work-life balance, and well-being) before the intervention, after the intervention, at a 4-week follow-up, and at a 6-month follow-up. The final sample included 373 participants (web-based intervention group: n*=*107, 28.7%; blended intervention group: n*=*129, 34.6%; and control group: n*=*137, 36.7%). Compliance was assessed as a secondary outcome.

**Results:**

The results of multilevel analyses were in line with our hypothesis that both training formats would improve psychological detachment, satisfaction with work-life balance, and well-being. We expected blended training to reinforce these effects, but blended training participants did not profit more from the intervention than web-based training participants. However, they reported to have had more social exchange, and blended training participants were more likely to adhere to the training.

**Conclusions:**

Both web-based and blended training are effective tools for improving the recovery, work-life balance, and well-being of workers with FWDs. Group sessions can increase the likelihood of participants actively participating in web-based modules and exercises.

**Trial Registration:**

German Clinical Trials Register DRKS00032721; https://drks.de/search/en/trial/DRKS00032721

## Introduction

### Background

An increasing share of workers have at least some autonomy in choosing their work times and locations. The COVID-19 pandemic further increased this number, which is expected to remain high. Flexible work designs (FWDs), such as flextime, telework, and mobile work, provide workers with temporal and spatial flexibility [1,2]. This can help meet both work and private life demands and thus reduce work-family conflicts [3,4]. It is also associated with physical health and reduced absenteeism [5]. However, workers with FWDs find it difficult to establish boundaries between work and private life [6]. This can impede the achievement of work-life balance, psychosomatic health, and recovery from work [7-9], which are essential for well-being [10].

Therefore, it is important to support workers in coping with these specific challenges of FWDs. Individual occupational web-based interventions can improve recovery, well-being, and work-life balance [11-14]. Web-based training has numerous advantages such as their flexible use for workers, high availability and accessibility to a large target group, and lower running costs. Thus, we developed a web-based intervention to promote self-regulation strategies in the context of FWDs, such as managing the boundaries between work and private life, detaching from work, establishing recovery periods, and self-organizing the workday.

However, we noticed that research tends to overlook the shortcomings of web-based interventions, such as high and easy dropout and feelings of isolation [15,16]. Common psychological theories, namely social identity theory and self-determination theory, state that social interactions and a sense of belonging to a group strengthen social support and motivation [17,18]. On the basis of these theoretical frameworks, we propose that a blended intervention, combining web-based self-training and face-to-face elements [15,19], should increase social support and adherence compared with web-based interventions. This should then reinforce the effectiveness of web-based training and further improve the outcomes. Thus, we also developed blended training for workers with FWDs, offering group sessions in addition to web-based modules. In this study, we aimed to examine the effectiveness of the general training approach (ie, irrespective of the training format) and compare whether a blended training format can address the shortcomings of an exclusively web-based training format, that is, strengthen social support as a resource and improve adherence.

This study contributes to the literature in several ways. First, there is little research comparing the effect of training formats in the work context, and past research that compared formats of occupational interventions has focused on comparing the effectiveness of face-to-face versus web-based interventions [14]. However, a more thorough investigation of these different training formats and their effects on outcomes is particularly important for practitioners who are to decide whether to offer web-based self-learning training or blended training, which incurs higher costs. This is even more important when there is less social interaction at the workplace, as people increasingly work in different locations, and social support is an important resource. We aimed to investigate whether individuals can profit more from an intervention with additional group sessions by comparing the effectiveness of web-based and blended training.

Second, based on social identity theory and self-determination theory, we aimed to empirically test the theoretical assumption that group interactions increase commitment, social support, and thus training effectiveness. In doing so, we integrated research on blended learning, which is mainly discussed in education psychology, and occupational intervention research, which is mainly discussed in occupational psychology. In particular, we combined research on learning settings and occupational interventions with the theoretical frameworks of group interactions. Moreover, we contributed to the discussion on how to reduce attrition in web-based interventions [16] and addressed the need to investigate whether perceived social support influences treatment adherence [20].

Third, we conducted this study as a randomized controlled trial with 4 measurement points, addressing the call for more randomized controlled trials on work-specific interventions [15,21,22]. Overall, this study contributed to the broader literature on occupational resource-oriented interventions.

### Effectiveness of an Intervention for Coping With FWDs

Workers with FWDs may face challenges, particularly those regarding their work-life balance, recovery, and well-being [2], such as difficulties in disengaging mentally from work, setting boundaries between work and private life, and recovering from work during breaks or leisure time. As FWDs usually come with fewer physical boundaries between work and private life, the blurring of role boundaries is likely [23]. This increases the likelihood of extended working time, taking fewer breaks, or being available during free time [2,8]. The shortening or interruption of periods between workdays can hinder recovery [9,24].

The training provided participants with several self-regulation strategies, that is, strategies to manage their behaviors, thoughts, and emotions [25], to address these specific challenges. They learned environmental (eg, establishing physical boundaries [26]) and cognitive-emotional (eg, practicing mindfulness [12]) segmentation strategies for creating and maintaining boundaries between work and private life. They learned respite strategies for enhancing their recovery during work breaks and after work, particularly strategies for promoting recovery experiences [27] and self-conducting rest periods [28]. Further, they learned specific self-regulation strategies for organizing their workdays and staying focused at work, which would facilitate mental disengagement after work.

These training strategies should enable workers to experience psychological detachment. This describes an essential recovery experience (ie, an off-job experience that is crucial for recovery) in which participants mentally disengage from work and its stressors and derive benefits for health, well-being, and work performance [29,30]. The training strategies should also enable workers to manage their boundaries, which should increase their satisfaction with work-life balance. Workers are satisfied with their work-life balance when they feel that they meet the multiple demands of work and family roles [31]. This should, then, improve well-being, which describes phenomena including “emotional responses, domain satisfactions, and global judgments of life satisfaction” [32]. Previous studies have shown that implementing training strategies enhances psychological detachment and improves satisfaction with work-life balance and well-being [12,27,33-36]. Hence, we expected that (hypothesis 1) after training, participants in both intervention groups (IGs) would report increased (1) psychological detachment, (2) satisfaction with work-life balance, and (3) well-being compared with the control group (CG) participants.

### The Importance of Intrinsic Motivation and Social Interaction for Training Effectiveness

In addition to its advantages, such as high availability and accessibility as well as lower running costs, web-based training has shortcomings, such as high and easy dropout and feelings of isolation [15]. Blended training combines the virtues of face-to-face and web-based approaches while compensating for their disadvantages [19,37]. In the blended training for this study, we combined individual web-based training with videoconferencing group sessions that focused on group-based methods (eg, group discussions, sharing challenges with FWDs, reflecting on experiences with training strategies together).

Group sessions may affect the motivation to engage in training, thus improving training effectiveness. Self-determination theory [18] proposes that people possess more or less self-determined motivation to engage in a particular behavior (eg, training exercises). The satisfaction of basic psychological needs facilitates intrinsic motivation, that is, self-determined behavioral engagement. These basic needs [38] include competence (ie, feeling effective and mastery), autonomy (ie, enacting self-endorsed behaviors), and relatedness (ie, belonging and feeling cared for by others). We expected the web-based training to satisfy the needs for competence and autonomy, and the additional group sessions to satisfy the need for relatedness. Hence, the intrinsic motivation to perform training exercises should be higher among blended training participants. Thus, we expected that (hypothesis 2) adherence and compliance rates would be higher for blended training participants than for web-based training participants.

Moreover, based on social identity theory, social interactions and a sense of belonging to a group can strengthen social support [17]. Mutual social support in an IG increases when training participants develop a sense of shared identity because they are members of a group; thus, group interaction processes result in improved employee health and well-being [17,39]. Moreover, based on the work-home resources model [40], social support perceived in the group sessions can be a contextual resource (ie, a resource located outside the self). Hence, strengthening social support as an important resource can have positive effects on outcomes in both the work and private life domains. As group interactions encourage the recognition that others also experience challenges with FWDs, we expected a sense of belonging and, hence, a shared social identity regarding FWDs and its management to arise in the group sessions. This can facilitate reciprocal validation and social support. The availability of social support can then improve training transfer and, thus, the immediate and long-term benefits of the training [15,41].

As previously stated, empirical evidence for these theoretical assumptions is scarce because most studies focus either on a specific workplace setting or on the comparison of blended or web-based versus face-to-face conditions (eg, the studies by Nortvig et al [42], Vallée et al [43], and Dunleavy et al [44]). A meta-analysis that compared blended learning with nonblended learning (eg, web-based learning or face-to-face learning) for health professions concluded that blended learning may be more effective than nonblended learning [37]. Moreover, shared team participation in a stress management intervention improved occupational self-efficacy [45], and web-based occupational interventions with guidance yielded better mental health [14]. In educational research, learning in small groups has been shown to reinforce students’ motivation and, thus, their achievements [46]. Further evidence stems from research on self-help support groups, showing that sharing mutual support and experiential knowledge in group interactions can help people manage personal challenges and change their behavior [47]. Thus, we expected the blended training to be more effective than the web-based training in teaching participants how to mentally detach from work, set boundaries between work and private life, and recover from work during breaks or leisure time. Therefore, we expected that (hypothesis 3) after training, the blended training participants would report a higher increase in (1) psychological detachment, (2) satisfaction with work-life balance, and (3) well-being than the web-based training participants.

## Methods

### Study Design and Procedure

From January to December 2021, we conducted a 3-armed randomized controlled trial with 2 IGs and a waitlist CG, with equal randomization across the groups. Because conducting the group training sessions required a lot of resources, there were 2 passes: one cohort (ie, web-based IG [IG-ON], blended IG [IG-BL], and CG) started in January, and the other started in May. The participants were aware of differing training start dates but were unaware of their assignment to one of the IGs or the waitlist CG (ie, single blind). However, they could not be blinded to their allocation to the web-based or blended training format because of the nature of format differences.

To recruit participants, we used a snowball sampling approach, email distribution lists, newsletters, professional networking websites, and magazine articles. We promoted the study as free training to help workers cope with the challenges of FWDs. The participants were aware that they would be randomly assigned to either web-based or blended training. The participants confirmed that they met the eligibility criteria (ie, they were at least 18 age years old, their jobs allowed them some flexibility, and they were willing to complete the training and all questionnaires) during registration via a website. We did not limit participation to a specific type of FWDs because FWDs can include different levels of flexibility, such as working in an office with flexible hours, telecommuting part time, or working remotely all the time. To complete the registration, the participants were required to sign an informed consent form and a data protection form. That is, the participants were provided with detailed study and privacy information and confirmed that they had read, understood, and accepted the information by checking a box. Because the registration process required internet access and a valid email address, computer and internet literacy were implied as eligibility criteria.

The participants completed a baseline (T0) questionnaire before we randomly allocated them to the waitlist CG, IG-ON, or IG-BL. As the dates for the blended training group sessions were set, the randomization of the participants into all 3 groups would have most likely resulted in higher attrition, as participants assigned to a specific blended group session might not have been able to attend. Hence, all the participants provided their time preferences for group sessions, knowing that these preferences would be relevant only when randomized to this particular group, when they registered. In an Excel (Microsoft Corporation) spreadsheet, a member of the author team generated a list of an equal number of group assignments and random numbers between 0 and 1. These were then sorted, resulting in a randomized list of group assignments, which were then matched to the list of participants. The participants who indicated that they did not have time on any of the available dates for the group sessions were randomized only between the IG-ON and waitlist CG (158/575, 27.5%). All other participants were randomized among all 3 experimental conditions (417/575, 72.5%).

After the IGs completed the training, we sent the postintervention time point (T1) questionnaire to all the participants. Four weeks later, we sent them the 4-week follow-up (T2) questionnaire. Then, the waitlist CG could access the training. Furthermore, we sent the IGs a 6-month follow-up (T3) questionnaire. We asked the participants to complete the questionnaires within 2 weeks. As an incentive for active participation, we offered participation certificates and information about project results.

### Intervention

All the participants across both training formats received the same web-based self-guided training. The 6-week web-based training was developed by the author team as a multicomponent self-regulation training with a toolkit of segmentation, mindfulness, self-organization, and recovery exercises to help participants cope with the specific challenges of FWDs and was previously evaluated in a randomized controlled trial [36]. The chosen exercises were proven to increase psychological detachment, satisfaction with work-life balance, and well-being [12,27,33-35]. The participants completed 6 weekly 45-minute training modules. They were made accessible on Thursdays, and we recommended engaging with them until the end of the weekend. In each module, we introduced the topic of focus for the week and then provided theoretical background information, self-reflection prompts, and practical exercises. Each module concluded with a self-regulation exercise based on self-regulation theories [25,48] to activate behavioral change, for example, mental contrasting with implementation intentions [49]. At the end of each module, we introduced a 5- to 10-minute daily task for the following 5 workdays to enhance training transfer and stimulate active learning [50]. We sent 3 emails or SMS text messages each week to remind the participants to perform the daily tasks. The intervention was designed as a toolkit in line with the positive-activity model [51], which emphasizes, among others, the promotion of person-activity fit, that is, the fit between person and activity characteristics. The participants were encouraged to keep practicing the exercises from their toolkit, which matched their preferences and needs and which they found the most helpful [52].

Multimedia Appendix 1 [53-72] provides a detailed overview of the intervention. Module 1 provides an overview of the aim and structure of the training. The participants formulated a participation goal to strengthen their motivation and commitment. As a daily task, the participants were to use an adapted version of the 54321 exercise [73]. Modules 2 and 3 focused on managing boundaries between work and private life based on boundary theory [74]. Module 2 introduced environmental segmentation strategies [26,33]. The daily task was to use 2 strategies for separating work and private life. Module 3 introduced mindfulness as a cognitive-emotional segmentation strategy [12]. The daily task was an adapted version of the 3-minute breathing exercise [12]. Module 4 introduced the self-regulation strategies of self–goal setting, self-monitoring, self-evaluation, and self-reward [25,48]. The daily task was to use these strategies to organize daily work. Module 5 focused on recovery through rest periods during off-job times and work breaks. The participants reflected on their recovery experiences [27] and learned a respite exercise [28]. The daily task was the respite exercise. Module 6 provided a summary of the previous modules, and the participants reflected on their personal and contextual resources [40]. The daily task was to think, in challenging situations, about the resources they would need and to reflect on a previous situation in which they had successfully used that resource.

The training was presented on a secure web-based platform programmed with a plug-in. It could be accessed via both desktop and mobile devices, although we recommended that participants not participate while on the move. Once the research team activated the account, the participants used their email address and a self-selected password to log in. Screenshots of the intervention are available in Multimedia Appendix 2.

In addition, the blended training participants were invited to participate in 3 group sessions. The groups included between 9 and 17 participants. The 3-hour videoconference group sessions took place on Thursdays or Fridays immediately before the start of the web-based training, that is, before module 1 (group session 1); after module 3 (group session 2); and after module 6 (group session 3). The group sessions were moderated by professional trainers. Approximately half of the group sessions were cofacilitated by a member of the author team to ensure consistency between the groups. The group sessions were designed to promote group interactions and social support (eg, exchanges in the group, in small groups, and in learning partnerships) and to consolidate what was learned in the web-based training. Although the sessions adhered to a standardized procedure, the group interactions gave participants the chance to cover aspects in greater depth according to their own needs based on the idea of optimizing the person-activity fit [51]. To further increase mutual support, we randomly assembled learning partnerships consisting of 3 or 4 participants and encouraged the participants to interact between group meetings.

### Participants

As only a small sample size of ≤50 participants at level 2 would lead to biased estimates in multilevel analyses [75], we conducted a power analysis that would allow an alternative examination of variances for a more conservative estimate of sample size. It revealed that 182 participants needed to be included to be able to detect an effect size of Cohen *d*=0.50 (η^²^_part_=0.06) between any 2 groups after treatment based on a power (1 – β) of 0.80 in a two-tailed test with α=.05, with α Bonferroni corrected for the number of examined variables. As a dropout rate of approximately 30% was assumed, a sample of at least 390 individuals was targeted (130 in each of the 2 IGs and the CG) to ensure sufficient power for the analyses.

**Figure 1:**
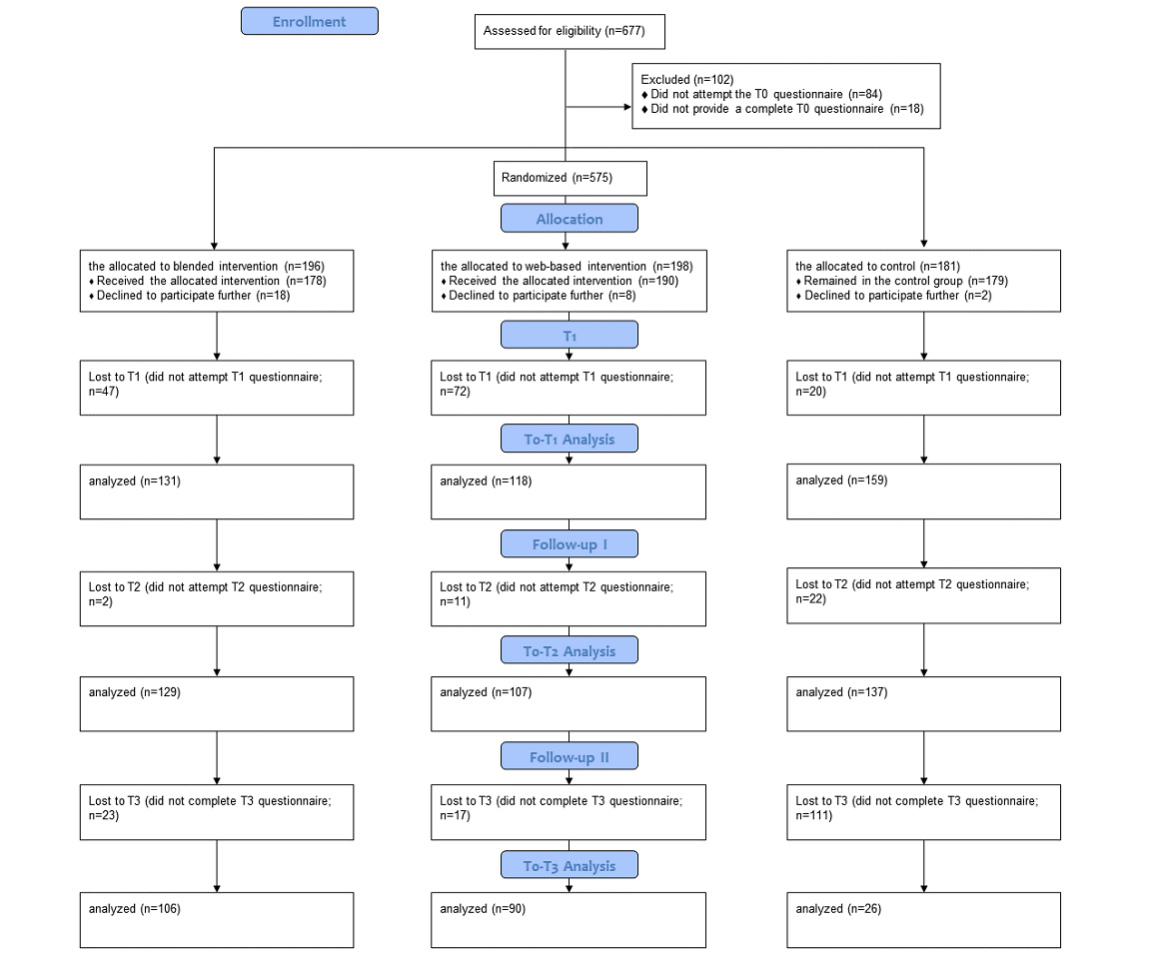
CONSORT (Consolidated Standards of Reporting Trials) flow diagram. T0: baseline; T1: postintervention time point; T2: 4-week follow-up; T3: 6-month follow-up.

The participants who completed the T0 questionnaire were randomly assigned to the IG-BL (196/575, 34.1%), IG-ON (198/575, 34.4%), or waitlist CG (181/575, 31.5%). The participants who dropped out at T1, that is, after the training (Figure 1), were more likely to hold leadership positions (*χ*^2^_1,582_=6.9; *P*=.009) and to work for longer hours (*F*_1,570_=10.06; *P*=.002) than nondropouts. The participants who did not complete the questionnaires at T2 and T3 were younger (*F*_1,580_=8.30, *P*=.004 and *F*_1,580_=13.85, *P*<.001) than those who completed the follow-up questionnaires. A higher dropout rate was observed in both IGs, particularly in the IG-ON, than in the CG; groups significantly differed at T1 (χ*^2^*_2,573_=42.0; *P<*.001) and T2 (χ*^2^*_2,573_=21.0; *P<*.001). Dropout rates also differed at T3 (χ*^2^*_2,573_=67.4; *P<*.001), when we observed a higher dropout rate in the CG, which had access to the web-based training by then.

**Figure 2:**
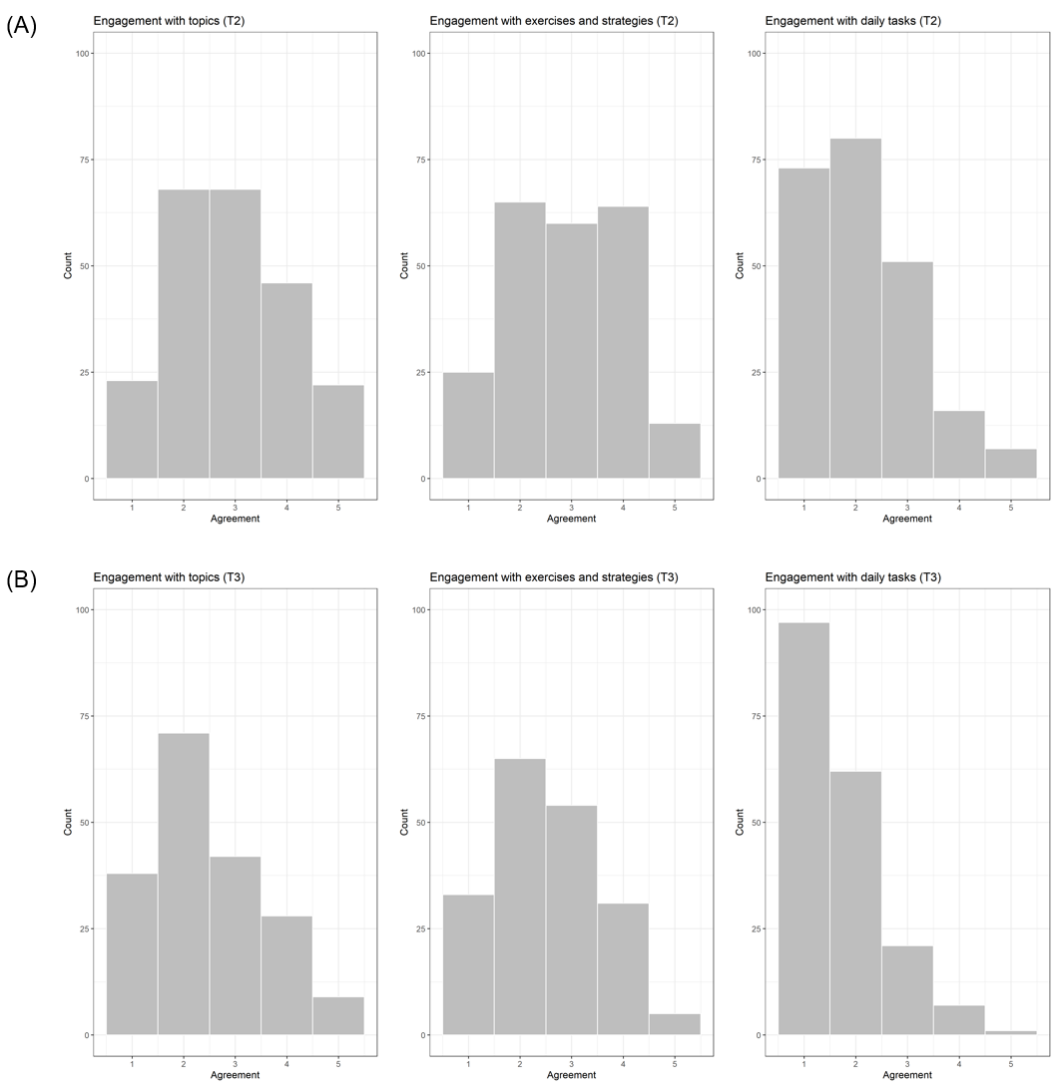
Posttraining engagement with the intervention content. (A) 4 weeks after training. (B) 6 months after training. T2: 4-week follow-up; T3: 6-month follow-up.

Table 1 shows how often the intervention participants who completed the T1 questionnaire reported practicing the daily task and the percentage of intervention participants who completed the modules at least partly. The blended participants reported having joined group sessions once (8/129, 6.2%), twice (35/129, 27.1%), thrice (85/129, 65.9%), or never (1/129, 0.8%). We also assessed posttraining compliance, that is, participants indicated on three 5-point Likert scales whether they were still engaging with the (1) topics of the web-based modules, (2) exercises and strategies, and (3) daily tasks after the end of the training (Figure 2). Overall, 4 weeks after the training (T2), 59.9% (136/227) at least somewhat agreed (ie, ≥3 on the respective scales) to still engage with the topics (IG-BL: 72/124, 58.1%; IG-ON: 64/103, 62.1%), 60.4% at least somewhat agreed (ie, ≥3 on the respective scales) to have continued to use the exercises and strategies to achieve the goals they set for themselves during the intervention (137/227, 60.4%; IG-BL: 76/124, 61.3%; IG-ON: 61/103, 59.2%), and 32.6% at least somewhat agreed (ie, ≥3 on the respective scales) to still practicing the daily tasks (74/227, 32.6%; IG-BL: 38/124, 30.6%; IG-ON: 36/103, 35%). Six months after the training (T3), 42% at least somewhat agreed to still engage with the topics (79/188, 42%; IG-BL: 42/104, 40.4%; IG-ON: 37/84, 44%), 47.9% at least somewhat agreed to have continued to use the exercises and strategies (90/188, 47.9%; IG-BL: 51/104, 49%; IG-ON: 39/84, 46%), and 15.4% (29/188) at least somewhat agreed to still practicing the daily tasks (IG-BL: 14/104, 13.5%; IG-ON: 15/84, 17.9%).

**Table 1:** Engagement with the training content at the postintervention time point.

		Use of daily task, mean (SD)	Module completion, n/N (%)
**Module 1**		3.17 (1.64)	232/237 (97.9)
	Blended format	3.20 (1.60)	125/127 (98.4)
	Web-based format	3.14 (1.69)	107/110 (97.3)
**Module 2**		3.44 (1.54)	231/237 (97.5)
	Blended format	3.59 (1.52)	126/127 (99.2)
	Web-based format	3.26 (1.55)	105/110 (95.5)
**Module 3**		2.69 (1.82)	226/237 (95.3)
	Blended format	2.90 (1.79)	122/127 (96.1)
	Web-based format	2.45 (1.83)	104/110 (95.5)
**Module 4**		2.55 (1.82)	212/237 (89.5)
	Blended format	2.71 (1.74)	116/127 (91.3)
	Web-based format	2.36 (1.66)	96/110 (87.3)
**Module 5**		2.45 (1.92)	203/237 (85.7)
	Blended format	2.60 (1.93)	112/127 (88.2)
	Web-based format	2.28 (1.90)	91/110 (82.7)
**Module 6**		1.85 (1.86)	185/237 (78.1)
	Blended format	1.97 (1.89)	103/127 (81.1)
	Web-based format	1.71 (1.82)	82/110 (74.5)

The final sample at T2 included 373 participants (IG-BL: n=129, 34.6%; IG-ON: n=107, 28.7%; CG: n=137, 36.7%) aged 23 to 64 (mean 46.40, SD 10.44) years; of them, 72.9% (n=272) were women, and 76.7% (n=286) held a university degree. Participants worked for an average of 39.34 (SD 9.64) hours per week; 92.8% (346/373) of the participants were employees (regular employees: 184/346, 53.1%; civil servants or public-sector employees: 137/346, 39.7%), 6.4% (24/373) were self-employed, and 0.8% (3/373) categorized themselves as having other types of employment; 25.2% (94/373) of the participants held a leadership position. The extent of temporal and spatial flexibility varied across our sample: 65.4% (244/373) could work flexible hours at least 5 days per week, and 96.8% (361/373) could work from home or in other locations for at least 1 day a week. The participants reported having the possibility to work from home or other locations on an average of 3.5 (SD 1.57) days per week and working flexible hours on an average of 4.21 (SD 1.65) days per week. The participants worked in various sectors, such as law, business, administration, science, teaching, and financial services. The participants across groups reported a higher preference for web-based training (mean 4.00, SD 1.06) than for blended training (mean 3.22, SD 1.31). Participants indicated their training preferences by rating their agreement with the statements “I prefer web-based training that I can work through independently” and “I prefer hybrid training that consists of web-based modules that I can work through independently and interactive training sessions where I meet other participants” on a 5-point Likert scale, 1=strongly disagree; 5=strongly agree. The study was conducted during the COVID-19-pandemic; 62.6% (233/373) of the participants stated that they worked from home more frequently in response to the pandemic, and 29% (108/373) of the participants had not worked from home before the pandemic. The participants in the IGs and CG had similar sociodemographic characteristics (Table 2), with 1 exception: the participants in the IGs were more likely to hold a leadership position (*χ*^2^_2,373_=8.4; *P*=.01) than the CG participants. Univariate ANOVAs showed that there were no significant differences in the study variables between the CG and IGs at T0.

**Table 2:** Means and SDs for sociodemographic characteristics at baseline (prequestionnaire).

Variable			IG-ON^a^ (n=107)		IG-BL^b^ (n=129)		CG^c^ (n=137)		Group differences				
									*F* test (*df*)		Chi-square (*df*)		*P* value
Age (years), mean (SD)			46.34 (10.33)		46.66 (10.63)		46.21 (10.42)		0.06 (2, 370)		N/A^d^		.94
Working hours, mean (SD)			39.75 (9.69)		40.43 (8.17)		37.99 (10.74)		2.27 (2, 369)		N/A		.11
**Individual preference for training format, mean (SD)**													
	Web-based training format	4.14 (1.12)		3.82 (1.06)		4.06 (1.00)		2.99 (2, 369)		N/A		.05	
	Blended training format	3.13 (1.39)		3.35 (1.26)		3.15 (1.30)		1.04 (2, 369)		N/A		.36	
	Spatial flexibility	3.34 (1.61)		3.69 (1.45)		3.44 (1.63)		1.64 (2, 370)		N/A		.20	
	Temporal flexibility	4.14 (1.76)		4.40 (1.57)		4.09 (1.62)		1.29 (2, 370)		N/A		.28	
**Gender, n (%)**									N/A		4.7 (4)		.32
	Women	73 (68.2)		93 (72.1)		106 (77.4)							
	Men	34 (31.8)		96 (27.9)		30 (21.9)							
	Nonbinary	0 (0)		0 (0)		1 (0.7)							
**Degree, n (%)**									N/A		8.1 (6)		.23
	Vocational training	11 (10.3)		18 (14)		22 (16.1)							
	Technical college degree	4 (3.7)		9 (7)		4 (2.9)							
	Bachelor’s degree	8 (7.5)		16 (12.4)		20 (14.6)							
	Master’s degree or equivalent	72 (67.3)		69 (53.5)		72 (52.6)							
	Doctorate	6 (5.6)		11 (8.5)		12 (8.8)							
	Habilitation	0 (0)		0 (0)		0 (0)							
	No professional qualification	1 (0.9)		0 (0)		0 (0)							
	Other	5 (4.7)		6 (4.7)		7 (5.1)							
**Employment status, n (%)**									N/A		4.6 (6)		.59
	Employees	59 (55.1)		64 (49.6)		75 (54.7)							
	Public-sector employee	37 (34.6)		55 (42.6)		56 (40.9)							
	Self-employed	10 (9.3)		9 (7)		5 (3.6)							
	Other	1 (0.9)		1 (0.8)		1 (0.7)							
**Leadership position, n (%)**									N/A		8.4 (2)		.01
	Yes	34 (31.8)		37 (28.7)		23 (16.8)							
	No	73 (68.2)		92 (71.3)		114 (83.2)							

^a^IG-ON: web-based intervention group.

^b^IG-BL: blended intervention group.

^c^CG: control group.

^d^N/A: not applicable.

### Measures

#### Overview

All variables were self-assessed in web-based questionnaires. We evaluated all variables, except demographics, at all 4 measurement points. We included compliance and manipulation checks as well as quantitative and qualitative feedback questions for training evaluation in the T1, T2, and T3 questionnaires. We used translation and back-translation procedures for items unavailable in German [76,77]. Unless otherwise indicated, we asked the participants to answer items referring to the preceding 2 weeks on a 5-point Likert scale (1=strongly disagree; 5=strongly agree).

#### Primary Outcome Measures

*Psychological detachment* from work during time off was assessed using a subscale of the Recovery Experience Questionnaire [29], which consisted of 4 items, for example, “after workhours, I distance myself from my work.” This scale showed very good reliability at all time points (T0: α=.89; T1: α=.89; T2: α=.91; and T3: α=.91).

*Satisfaction with work-life balance* was assessed using 4 items from the Satisfaction With Work-Family Balance Scale [31] that Michel et al [12] adapted to focus on private life rather than family life, for example, “How satisfied are you with how well your work life and your private life fit together?” The participants answered on a 5-point scale (1=very dissatisfied; 5=very satisfied). This scale demonstrated very good reliability at all measurement points (T0: α=.91; T1: α=.90; T2: α=.92; and T3: α=.93).

*Positive affective well-being* was measured using the 5-item World Health Organization Well-Being Index [78]. The participants rated all items, for example, “over the last two weeks, I felt cheerful and in good spirits,” on a 6-point frequency scale (1=at no time; 6=all the time). This scale showed good reliability at all time points (T0: α=.88; T1: α=.90; T2: α=.90, and T3: α=.92).

#### Manipulation Checks

##### Learning About Strategies

As a manipulation check for the intervention, we developed and used a 5-item scale to assess learning about strategies to cope with FWDs. (Following Hahn et al [27], using general questions seems appropriate because the participants are not asked to adopt specific behaviors but rather encouraged to identify and choose strategies that are helpful for them. Hence, the participants could show a wide range of behaviors after the training.) We asked the participants whether they had learned anything about strategies to cope with the challenges of FWDs during the last 6 weeks. The items were “in the last six weeks, I learned...,” “...how to set boundaries between work and private life,” “...how to detach from work,” “...how to improve my self-organization,” “...how to recover,” and “...how to become aware of my resources.” This scale showed very good reliability (α=.92).

##### Social Exchange

As a manipulation check for the blended training, we assessed the *experiential knowledge provided* (eg, “I shared my feelings regarding my temporal and spatial flexibility”) and *emotional support received* (eg, “other people listened carefully when I talked about managing my temporal and spatial flexibility”) with the respective 3-item subscales of the Self-Help Support Group Social Exchange Scales [47], adapted to focus on the context of FWDs. Items were rated on a 5-point frequency scale (1=rarely or never; 5=often or always). The subscales showed very good reliability (experiential knowledge provided: α=.90; emotional support received: α=.89).

#### Secondary Outcome Measures

To measure compliance, we asked the following question with regard to each of the 6 training modules at T1: “have you worked through the module?” The participants answered on a 5-point scale (1=no; 5=yes, completely). We also asked, “on how many days during the week after you learned about the daily exercise did you practice it?” The participants could indicate “none” and up to “more than five days.” In addition, trainers filled in an attendance list to document how often the blended training participants joined group sessions.

### Analysis Strategy

All analyses are reported according to the extended CONSORT (Consolidated Standards of Reporting Trials) eHealth checklist [79]. To examine adherence and compliance among the training participants, we performed logistic regressions to analyze the effects of group membership on the likelihood that participants would drop out and that participants would be compliant. To test the hypotheses regarding intervention effectiveness, we conducted multilevel regression analyses with measurement occasions (level 1) nested within participants (level 2). The calculation of intraclass correlation coefficients suggested that substantial amounts of variance could be attributed to the between-person level of analysis in all outcome variables (psychological detachment: 63.4%; satisfaction with work-life balance: 65.5%; well-being: 66.8%), justifying the use of multilevel analyses. As multilevel modeling does not require balanced data [80], dropout is not a concern. To further meet the requirements for an intention-to-treat analysis, we included the data of all the participants regardless of their compliance. We performed analyses in R [81] using the R package *lme4* [82]. To test intervention effectiveness, our linear mixed model included fixed effects of group, time, and their interaction effect and a random effect of participants. Time was dummy coded (preintervention time point vs T1 and preintervention time vs follow-up [83]). Group was contrast coded (CG vs both IGs and IG-ON vs IG-BL [80]). Mean centering was not necessary because the multilevel model only contained dummy variables as independent variables [84].

### Ethical Considerations

This study was granted ethical approval from the ethics committee of the Federal Institute for Occupational Safety and Health in Germany (032_2019_Michel). Owing to several restrictions (eg, high degree of uncertainty as to whether we would be able to conduct the study as planned due to COVID-19 restrictions), we were unable to prepare a properly detailed protocol for a preregistration. However, we registered the study retrospectively after it ended in the German Clinical Trials Register (registration number: DRKS00032721).

## Results

### Overview

**Figure 3:**
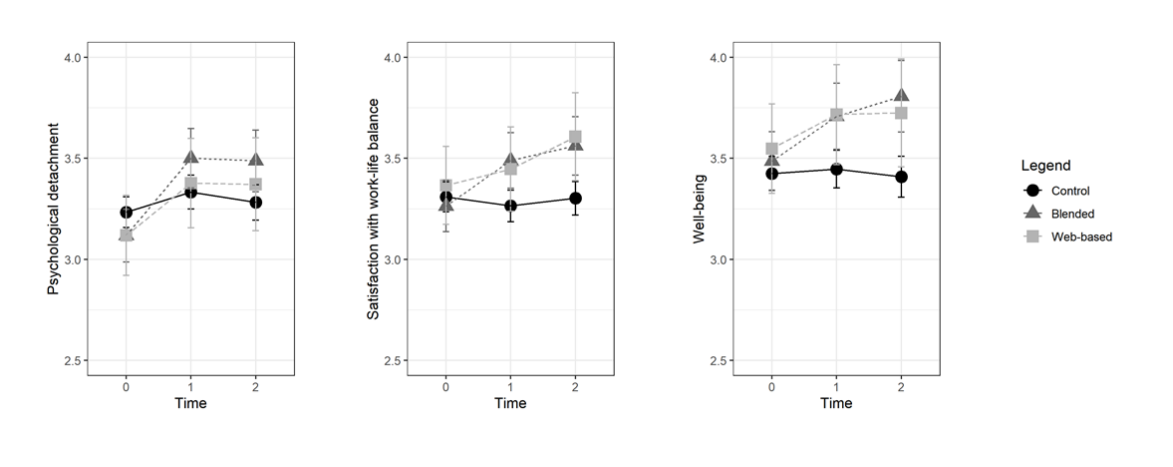
Means of the outcome variables for the intervention groups and control group at baseline, postintervention time point, and 4-week follow-up. The error bars indicate the lower and upper bounds of the CI for the predicted values.

Table 3 provides descriptive information for the IGs and waitlist CG at all measurement points. Figure 3 shows the mean scores of all the groups.

**Table 3:** Means and SDs for the outcome variables at baseline (T0), postintervention time point (T1), 4-week follow-up (T2), and 6-month follow-up (T3).

Variable	T0 (n=408), mean (SD)				T1 (n=408), mean (SD)				T2 (n=373), mean (SD)				T3 (n=222), mean (SD)	
	IG-ON^a^	IG-BL^b^	CG^c^	IG-ON		IG-BL	CG	IG-ON		IG-BL	CG	IG-ON		IG-BL
Psychological detachment	3.13 (0.89)	2.96 (0.96)	3.23 (0.90)	3.36 (0.80)		3.41 (0.87)	3.34 (0.90)	3.41 (0.88)		3.45 (0.91)	3.31 (0.93)	3.40 (0.80)		3.36 (0.95)
Satisfaction with work-life balance	3.35 (0.92)	3.20 (0.94)	3.32 (0.92)	3.43 (0.83)		3.45 (0.80)	3.28 (0.88)	3.67 (0.75)		3.57 (0.85)	3.33 (0.91)	3.42 (0.82)		3.53 (0.89)
Well-being	3.57 (1.03)	3.52 (0.97)	3.42 (1.08)	3.72 (0.97)		3.72 (0.94)	3.45 (1.12)	3.82 (0.99)		3.85 (1.04)	3.39 (1.08)	3.69 (1.08)		3.67 (1.09)

^a^IG-ON: web-based intervention group.

^b^IG-BL: blended intervention group.

^c^CG: control group.

### Manipulation Checks

As a manipulation check for both interventions, we examined whether the intervention participants reported having learned anything about strategies to cope with the challenges of FWDs during the last 6 weeks. ANOVA yielded significant variation among the groups (*F*_2,392_=169.5; *P*<.001). A post hoc Tukey test showed that the IGs differed significantly from the waitlist CG (*P*<.001); the IG-BL was not significantly different from the IG-ON. Thus, both IGs learned strategies to cope with the challenges of FWDs, which showed the effect of the intervention.

As a manipulation check for the different training formats, we tested whether the blended training participants reported more social exchanges than the web-based training participants. ANOVAs yielded significant variation among the groups for experiential knowledge (*F*_2,392_=21.85; *P*<.001) and emotional support (*F*_2,392_=16.91; *P*<.001). Post hoc Tukey tests showed that the blended training participants differed significantly from both the CG and web-based training participants (*P*<.001). Thus, the blended training participants experienced increased sharing of experiential knowledge and emotional support, affirming the effect of the blended training.

### General Effectiveness of the Intervention

Table 4 summarizes all the coefficients for the multilevel analyses. For psychological detachment, satisfaction with work-life balance, and well-being, the results showed a significant intervention effect (ie, IG vs CG × time interaction) at T1 and T2, supporting hypothesis 1, which assumed that both training formats would improve psychological detachment, satisfaction with work-life balance, and well-being. That is, both IGs reported higher scores than the CG, both at T1 and at T2. Moreover, regarding all 3 outcomes, time had a main effect both for preintervention-postintervention and preintervention-follow-up comparisons, and group had no significant main effect.

**Table 4:** Results of the multilevel models for all outcomes (number of observations included in the analysis; n=1332)^a^.

Outcome and predictor			B (SE; 95% CI)		*t* value
**Psychological detachment**					
	Intercept	3.16^b^ (0.04; 3.08 to 3.23)		81.64	
	IG^c^ vs CG^d^	−0.08 (0.06; −0.19 to 0.03)		−1.39	
	IGBT^e^ vs IGOT^f^	−0.00 (0.09; −0.18 to 0.18)		−0.01	
	Time 1	0.25^b^ (0.04; 0.18 to 0.32)		6.80	
	Time 2	0.22^b^ (0.04; 0.15 to 0.30)		5.61	
	IG vs CG × time 1	0.15^g^ (0.05; 0.05 to 0.24)		2.98	
	IG vs CG × time 2	0.18^g^ (0.06; 0.07 to 0.28)		3.19	
	IGBT vs IGOT × time 1	0.12 (0.09; −0.06 to 0.30)		1.35	
	IGBT vs IGOT × time 2	0.12 (0.10; −0.08 to 0.31)		1.17	
**Satisfaction with work-life balance**					
	Intercept	3.31^b^ (0.04; 3.24 to 3.39)		87.70	
	IG vs CG	0.00 (0.05; −0.10 to 0.11)		0.07	
	IGBT vs IGOT	−0.10 (0.09; −0.28 to 0.08)		−1.12	
	Time 1	0.09^g^ (0.03; 0.02 to 0.15)		2.58	
	Time 2	0.18^b^ (0.04; 0.10 to 0.25)		4.54	
	IG vs CG × time 1	0.13^g^ (0.05; 0.04 to 0.22)		2.87	
	IG vs CG × time 2	0.18^b^ (0.05; 0.08 to 0.29)		3.42	
	IGBT vs IGOT × time 1	0.15 (0.09; −0.02 to 0.31)		1.70	
	IGBT vs IGOT × time 2	0.06 (0.10; −0.13 to 0.25)		0.58	
**Well-being**					
	Intercept	3.49^b^ (0.04; 3.40 to 3.57)		80.77	
	IG vs CG	0.06 (0.06; −0.06 to 0.18)		0.99	
	IGBT vs IGOT	−0.06 (0.10; −0.26 to 0.14)		−0.58	
	Time 1	0.14^b^ (0.04; 0.06 to 0.21)		3.46	
	Time 2	0.16^b^ (0.05; 0.07 to 0.25)		3.48	
	IG vs CG × time 1	0.12^h^ (0.05; 0.01 to 0.22)		2.14	
	IG vs CG × time 2	0.18^g^ (0.06; 0.05 to 0.30)		2.78	
	IGBT vs IGOT × time 1	0.05 (0.10; −0.14 to 0.25)		0.52	
	IGBT vs IGOT × time 2	0.14 (0.12; −0.08 to 0.37)		1.24	

^a^Zero is not included in the reported CIs if the lower and upper bounds of the CI have the same sign. In the reported CIs, numbers not equal to zero would appear if more decimal places were reported.

^b^*P*<.001.

^c^IG: intervention group.

^d^CG: control group.

^e^IGBT: blended intervention group.

^f^IGOT: web-based intervention group.

^g^*P*<.01.

^h^*P*<.05.

### Adherence and Compliance

Hypothesis 2 proposed that the blended training participants would be more adherent and compliant than the web-based training participants. Regarding the difference in dropout between the training formats, the web-based training participants were not more likely to drop out (80/198, 40.4%) than the blended training participants (65/196, 33.2%) at T1 (odds ratio [OR] 1.37, 95% CI 0.91-2.06) and T3 (web-based: 108/198, 54.5%; blended: 90/196, 45.9%; OR 1.41, 95% CI 0.95-2.10). At T2, the web-based training participants were twice as likely to drop out (91/198, 46%) as the blended training participants (67/196, 34.2%; OR 1.64, 95% CI 1.09-2.46). Regarding the difference in compliance between the training formats, the blended training participants were 17 times more likely to be compliant (142/143, 99.3%) than the web-based training participants (98/110, 89.1%; OR17.39, 95% CI 2.23-135.87). Thus, hypothesis 2 was partially supported.

### Differences Between the IGs

Hypothesis 3 proposes that the blended training participants would profit more from the intervention than the web-based training participants in terms of psychological detachment, satisfaction with work-life balance, and well-being. Analyses revealed that the intervention effects did not differ between the IGs at T1 and at T2 (ie, IG-BL vs IG-ON × time interaction). That is, the blended training participants reported scores that were not significantly higher than those of the web-based training participants, both at T1 and at T2. Thus, we rejected hypothesis 3.

### Additional Analyses

To explore the long-term effectiveness of the intervention, we analyzed the main effect of time at T3 for both IGs in multilevel regression analyses, which was significant for psychological detachment (B=0.24, SE 0.08; *t*=2.84; 95% CI 0.07-0.40) but not for satisfaction with work-life balance (B=0.05, SE 0.08; *t*=0.68; 95% CI −0.10 to 0.20) and well-being (B=0.09, SE 0.10; *t*=0.90; 95% CI −0.11 to 0.29). To explore differential long-term effects, we analyzed intervention effects (ie, IG-BL vs IG-ON × time interaction) between the IGs at T3. These analyses did not reveal differences in intervention effects at T3 for psychological detachment (B=0.06, SE 0.11; *t*=.55; 95% CI −0.16 to 0.29) or well-being (B=0.04, SE 0.14; *t*=0.30; 95% CI −0.23 to 0.31). However, there was a significant difference between the training formats regarding satisfaction with work-life balance at T3 (B=0.21, SE 0.10; *t*=2.02; 95% CI 0.01-0.41) such that the blended training participants profited more.

As a robustness check, we excluded the training participants from multilevel regression analyses who reported only rudimentary or no compliance to the training modules or practiced the daily tasks fewer than 2 days per week (web-based and blended training) and attended fewer than 2 group meetings (blended training), resulting in a per-protocol analysis. The results held for general effectiveness at T1 and T2 for all outcomes and again revealed no difference between the training formats. They were also similar for the long-term effectiveness of the intervention at T3, except that there was no longer a significant difference between the training formats regarding satisfaction with work-life balance at T3. As a further robustness check, we conducted all multilevel analyses with time as a numeric variable [80]. The results held for both general and differential intervention effectiveness. These results add to the robustness of the findings regarding posttraining measures and T2 measures. Only when those who did not regularly engage with the web-based modules and exercises and attended only 1 or no group meetings were included in the analyses (intention-to-treat) were the blended training participants more satisfied with their work-life balance 6 months after the training ended than the web-based training participants.

As preliminary analyses revealed that the participants in the IGs were more likely to hold a leadership position than those in the CG, we conducted multilevel regression analyses with leadership as an additional predictor. The results held for the general effectiveness of the intervention (ie, IG vs CG × time) at T1 and T2 for all outcomes. Intervention effects between the IGs at T1 and T2 (ie, IG-BL vs IG-ON × time interaction) remained insignificant. That is, the effects of general and differential effectiveness were robust when adjusting for whether the participants held a leadership position.

We conducted subgroup multilevel regression analyses to explore the effect of employment status on training effectiveness (Multimedia Appendix 3). With employment status included in the analyses, the results of general effectiveness held for all outcomes. Moreover, the results indicated the differential effectiveness of the training formats regarding satisfaction with work-life balance at T1. Regarding 3-way interactions, a significant effect was found for public-sector employees (IG vs CG × time × employment status) regarding satisfaction with work-life balance at T1. In addition, significant 3-way-interactions were found for other types of employment (IG-BL vs IG-ON × time × employment status) for satisfaction with work-life balance at T1 and for well-being at T2. That is, general training effectiveness (ie, intervention effectiveness irrespective of the training format) regarding satisfaction with work-life balance immediately after the training was less nuanced for public-sector employees. As the subgroup of those with other types of employment consisted of only 3 people, these results are of limited value and should not be interpreted.

To explore whether the participants who reported low social support at T0 profited more from the blended training, we conducted multilevel regression analyses with social support as an additional moderator. Social support was measured using the subscale for perceived available instrumental support of the Berliner Social-Support Scales [85], which consisted of items such as “when I am worried, there is someone who helps me.” This scale showed good reliability (T0: α=.90). The respective interaction (IG-BL _vs_ IG-ON × time × social support) was not significant for psychological detachment at T1 (B=−0.03, SE 0.11; *t*=−0.24; 95% CI −0.25 to 0.19) and T2 (B=−0.11, SE 0.13; *t*=−0.83; 95% CI −0.36 to 0.14), satisfaction with work-life balance at T1 (B=−0.01, SE 0.11; *t*=−0.08; 95% CI −0.21 to 0.20) and T2 (B=0.06, SE 0.12; *t*=0.49; 95% CI −0.18 to 0.30), and well-being at T1 (B=0.15, SE 0.12; *t*=1.19; 95% CI −0.09 to 0.39) and T2 (B=0.04, SE 0.15; *t*=0.25; 95% CI −0.25 to 0.33).

## Discussion

### Summary of Results

Workers with FWDs face specific challenges regarding their work-life balance, recovery from work, and well-being [2]. First, we aimed to examine the effectiveness of our general training approach by teaching participants to cope with these particular challenges using self-regulation strategies. Second, we aimed to compare the effectiveness of web-based and blended training formats. On the basis of social identity theory and self-determination theory, we specifically expected social interactions within group sessions and a sense of belonging to strengthen both social exchange and motivation [17,18], addressing the main shortcomings of a web-based format, the lack of social interaction and high dropout. Moreover, we expected more social exchange and higher motivation to increase training effectiveness. Multilevel analyses supported the overall effectiveness of the training approach. Although there was no difference in effectiveness between the training formats, the blended training participants were more compliant.

In line with our hypotheses, multilevel analyses showed that the training (both web-based and blended formats) improved psychological detachment, satisfaction with work-life balance, and well-being compared with a waitlist CG. This shows that our 6-week web-based training offers strategies that help workers cope with the specific challenges of FWDs. Specifically, it provides participants with segmentation strategies that help them set boundaries between life domains. They learn respite strategies that help them enhance their recovery during work breaks and after work. Further, they learn strategies that help them self-organize their workdays. In addition to the robustness of these findings, they held in a per-protocol analysis, that is, when excluding those who were not compliant with the training protocol and when including employment status in the analyses. This is in line with research showing that individual web-based interventions can be effective in teaching activities for promoting recovery from work, work-life balance, and well-being [12,27,33-35] and provide self-regulation strategies to help overcome the challenges associated with FWDs [86].

In addition, we found that adherence and compliance were in some ways higher among the blended training participants, partly supporting hypothesis 2: 4 weeks after the training, the web-based training participants were twice as likely to drop out as the blended training participants. Moreover, the blended training participants were 17 times more likely to be compliant than the web-based training participants, that is, with completing training the modules at least partially and practicing the daily tasks for at least 2 days per week. These results indicate that interacting with peers in group sessions, in addition to the web-based modules, significantly affected social exchange as well as the motivation and commitment of participants. Feedback from the blended training participants reflects these results; some felt that regular meetings helped them follow through with the training. This is in line with the argument based on self-determination theory that the satisfaction of relatedness as a basic need in the blended training would increase intrinsic motivation. This contributes to nascent efforts to identify factors that may increase adherence, building on findings showing feedback and content-focused or adherence-focused guidance [87,88], motivational and volitional processes [20], and engaging content and time efficiency [89] as such factors. However, these findings should not be generalized to any web-based versus blended training, as the positive effects on compliance may be influenced by the specific training content. That is, in particular, the sharing of strategies and exercises to overcome the challenges of FWDs may have triggered compliance because of their relevance to participants’ daily routines. The pandemic context may also have reinforced these effects, as many people had to work more independently than before and may have had problems keeping up with yet another web-based service.

The manipulation in the IG-BL was successful, as we found social exchange (ie, experiential knowledge provided and emotional support received) to be higher among the blended training participants than among the web-based training participants. This is in line with the argument based on social identity theory that group interactions in blended training increase social exchange. However, intervention effectiveness did not differ between the IGs, neither directly after the training nor 4 weeks later. Group sessions did not reinforce the effects of the web-based training, although they seemed to have increased social exchange and motivation. These findings are inconsistent with the assumption drawn from social identity theory that increased social exchange through group interactions would improve the effectiveness of the blended training. Although this is not in line with our hypotheses, there are some studies that point toward a similar direction. For example, a study that compared web-based learning, blended learning, and face-to-face learning did not find any effect of training mode on knowledge or confidence [90]. In the educational sciences, a meta-analysis did not find the expected effect for the combination of distance education and face-to-face instruction compared with distance education; however, they could include only a few effect sizes [91]. Nevertheless, as there have been few studies with inconclusive findings on this matter, this is an area in need of research attention. The need for further research is underlined by findings indicating the differential effectiveness of the training formats on satisfaction with work-life balance immediately after training when employment status (ie, employed in the private or public sector or self-employed) was included in the analyses, suggesting that more research on differential effects is needed. In addition, the results indicated a potentially higher long-term effect of blended training on work-life balance at 6 months after training; however, as this effect vanished in a per-protocol analysis, it should be interpreted with caution.

It is likely that the blended training participants built a sense of belonging to a group and relatedness by sharing their experiences with FWDs and getting to know each other. Moreover, they may have had perceived social pressure to work through the modules and implement the exercises because they knew that they would talk about them with their group. This is in line with the reasoning that the social influence of a group can affect people’s commitment and motivation [92]. Articulating one’s own challenges with FWDs and desires for change within group sessions may have increased intrinsic motivation to implement training strategies even more. However, increased motivation did not affect the training outcomes. One explanation for these unexpected results regarding group differences is that there might have been a selection effect: in the web-based training, those who did not find the training helpful likely dropped out, whereas in the blended training, participants felt committed to continuing the training, even if they did not find it suitable for themselves. This could have led to training effects being overestimated in one group and underestimated in the other.

### Limitations and Implications for Future Research

This study has several strengths, such as the randomized controlled trial design and 2 long-term follow-up questionnaires. This provides robust evidence for our research questions, addressing the need for more randomized controlled trials on work-specific interventions [21,22].

However, this study also has limitations. In our training, we combined multiple strategies that help address different challenges in the context of FWDs. We believe that this comprehensive approach is of high practical relevance because of the multifaceted nature of FWD challenges and the heterogeneity of the emphasis people place on certain challenges. When all strategies are learned in the first place, it becomes more likely to find personally helpful strategies to cope with FWDs. Moreover, the focus of this study was on comparing web-based and blended training. Nevertheless, future research could question the superiority of one of these components or their combination and test the effects of separate and combined components against complete training. Alternatively, weekly diaries and growth curve models would allow the evaluation of the effectiveness of specific modules.

Further, women and participants with high education were overrepresented in this study because they selected themselves. That is, the results of this study are not representative. Although the participants with leadership positions were not evenly distributed across the groups, controlling for leadership did not affect the robustness of the results. However, interventions are most successful when participants self-select into the study [93], probably because they experience a high need for training. Face-to-face group sessions did not take place in person but took place via videoconferencing because of the pandemic situation. However, the framing of additional videoconferences as blended training is common in intervention research [90]. Moreover, based on media richness theory, videoconferencing can be considered a rich medium, being almost as rich as face-to-face communication [94]. This is also reflected in web-based support groups providing similar helping techniques to those provided by face-to-face support groups [95]. Hence, we expect similar underlying social processes and outcomes.

We only assessed compliance via self-report because, owing to data protection requirements, we were not able to link participants’ questionnaires with their module engagement. Future studies could incorporate objective measures such as module completion status to measure actual compliance. To examine whether alternative interventions are similarly or more effective and whether participants’ expectations regarding their participation served as a demand characteristic evoking hypothesis-conforming behavior [96], future research could add a further CG that receives an alternative or placebo intervention [21]. We argued that motivation to engage with training may play an important role in explaining training effectiveness. Future research could explore this assumption in more detail and, to do so, measure motivation with specific scales. Moreover, we measured social exchange only as a manipulation check and, hence, only after training. Future research could include social exchange measures from the beginning to allow for modeling the change over time. In addition, future research could also assess the type of work (eg, interaction work and work on a PC) and analyze whether the general training approach and specific training formats are more suitable for certain types of work.

Finally, because of the conduct of group sessions, complete randomization was not feasible. However, this is a common approach in training programs that require the presence of participants [97].

### Practical Implications

Workers with FWDs face specific challenges, such as with maintaining boundaries between work and private life, detaching from work, establishing recovery periods, and self-organizing their workday. In this study, we show that training that teaches self-regulation strategies, namely environmental and cognitive-emotional segmentation strategies, recovery strategies, and self-organization strategies, helps participants improve psychological detachment, satisfaction with work-life balance, and well-being. Hence, we recommend that interventions for workers with FWDs teach such self-regulation strategies. To support workers with FWDs in terms of their psychological detachment, satisfaction with work-life balance, and well-being, occupational health managers, HR managers, or supervisors can offer such a self-regulation intervention. To do so, they may use a self-guided learning manual based on the web-based training (eg, the English version of the German self-guided learning manual [98]), available upon request from the authors. Alternatively, they may develop a similar intervention based on the results of this study.

To determine whether conducting blended training is worth the additional time, effort, and cost, we compared a web-based training group with a blended training group. We found the intervention to be effective for all participants regardless of the training format. However, we found that the blended format was beneficial for participants’ adherence and commitment, supposedly because they experienced more social interaction. This is important, as a key shortcoming of web-based training is high dropout. Accompanying group meetings can increase the likelihood of training adherence. This underlines the importance of sharing experiences with others to follow through and truly engage with web-based training, which is in line with research showing that people who experience a sense of belonging to a group are more likely to coordinate with the goal pursuit of others in the group [99]. Moreover, blended training could address the danger of social isolation; people have fewer social interactions and perceive less social support when they work in different locations, and social isolation is one of the greatest disadvantages workers perceive with mobile work [100].

Thus, when practitioners decide that it is worth the increased effort of blended training to strengthen social exchange and commitment, they should encourage group interactions accompanying web-based training, for example, by offering group sessions or regular meetings. Moreover, employees who participate in web-based training can share their experiences and goals for participation with others to increase their own commitment to follow through with the training. Importantly, these applications would then differ slightly from the group sessions evaluated in this study, limiting the transferability of the study results. Individual interventions can serve as a valuable addition to human resource practices and policies. However, they should always be considered as an addition to appropriate working conditions. These include, for example, support from supervisors and peers and corporate agreements on telework [7,101].

### Conclusions

In this study, we showed that an intervention that aims to promote self-regulation strategies to cope with FWDs, such as managing boundaries, recovering from work, and self-organizing workdays, is effective. In a randomized controlled trial, multilevel analyses showed that participation in the intervention improved work-life balance, recovery, and well-being. The training was effective regardless of its format, which was either web-based or blended. However, adherence 4 weeks after training and compliance were higher among the blended training participants. The share of workers with temporal and spatial flexibility is expected to remain high in the future. Web-based self-regulation intervention can be a helpful tool in supporting workers to cope with the specific challenges of FWDs. Moreover, group sessions accompanying web-based training can strengthen compliance.

## Appendix

Multimedia Appendix 1 Intervention overview.

Multimedia Appendix 2 Screenshots of the intervention.

Multimedia Appendix 3 Additional analyses.

Multimedia Appendix 4 CONSORT-EHEALTH checklist.

## Abbreviations

CG: control group
CONSORT: Consolidated Standards of Reporting Trials
FWD: flexible work design
IG: intervention group
IG-BL: blended intervention group
IG-ON: web-based intervention group
OR: odds ratio
T0: baseline
T1: postintervention time point
T2: 4-week follow-up
T3: 6-month follow-up
