# Supplementary material for: Comparing Web-Based and Blended Training for Coping With Challenges of Flexible Work Designs: Randomized Controlled Trial
Source: J Med Internet Res. 2023 Dec 19;25:e42510. doi: 10.2196/42510 (PMC10762610; doi:10.2196/42510)
Supplement: Multimedia Appendix 1 [file jmir_v25i1e42510_app1.docx]

**Multimedia Appendix 1**

*Intervention Overview*

| Modules | Intervention Elements |
| --- | --- |
|  |  |
| Group Session 1  (only for participants of the blended training) | - adapted version [12] of the 3-minute breathing exercise [53, 54] - initiate social interaction (experience yourself as part of the group, tell about yourself) - sharing preparations or rituals for the individual work on the online modules – with a focus on listening to others – in small groups - introduction to central concepts of the online training: setting goals, reflecting resources - random assignment to learning partnerships: mutual mentoring and support, motivation - exchange in learning partnerships to get to know each other (training motivation, goals and challenges in the context of FWD) |
| Module 1: *Working flexibly – healthfully and happy* | - formulate a training goal to strengthen motivation and commitment - reflect on goals achieved in the past to activate personal resources - set specific, measurable, achievable, relevant, time-bound (SMART) participation goals [55] to assure regular participation - reflect on personal training motivation using mental contrasting with implementation intentions [MCII; 49] to strengthen goal striving - reflect on previously used strategies to activate personal resources for coping with FWD   *Daily task*: adapted version of the 54321 exercise [73]: When you find yourself ruminating about work, focus on the moment instead.  *Self-regulatory element*: set specific participation goals and use MCII to set the overall training goal |
| Module 2: *Finding personal balance* | - study theoretical background regarding conflict between work and nonwork roles [56], boundary theory, and the segmentation-integration continuum [74] in the context of FWD - learn the importance of establishing and managing boundaries aligned with segmentation preferences for separating or integrating work and nonwork domains [101] and how integrators can profit from segmentation strategies in certain times and situations - reflect on actual and preferred separation or integration between work and nonwork domains - introduce physical, temporal, behavioral, and technological boundary management tactics [26] and transition rituals [74] to separate work from nonwork   *Daily task*: Use two specific strategies for separating work and nonwork domains.  *Self-regulatory element*: set specific goals and apply MCII for the daily task |
| Module 3: *Switching off from work – setting boundaries* | - learn about detachment practices that enhance health and well-being [e.g., 57] - reflect on activities that block out thoughts about work, based on recovery training from Hahn et al. [27] - introduce the two-component model of mindfulness [58] and discuss its positive effects on stress and well-being [e.g., 59-62] - focus on self-regulation of attention, the first component of mindfulness   - an audio exercise guides participants to focus on their breathing, and to draw their attention back to their breath when minds wander [53, 54, 63]   - participants learn to distance themselves from work-related thoughts by turning attention to the current moment and away from past or future-orientated cognitions - focus on mindful orientation to experience, the second component of mindfulness   - an audio exercise shows how incidents evoke subjective viewpoints [53, 54, 63, 64]   - participants learn that being mindfully oriented helps them escape undesired work-related thoughts and feelings   *Daily task*: Use the adapted version [12] of the 3-minute breathing exercise [53, 54] to support transition to private life after work or to distance from work-related thoughts and feelings  *Self-regulatory element*: setting specific goals and applying MCII for daily tasks |
| Group Session 2  (only for participants of the blended training) | - adapted version [12] of the 3-minute breathing exercise [53, 54] - short review of modules 1-3 - sharing about positive experiences of the past three weeks (e.g., small successes, helpful strategies or resources within in the online training, learning partnership, or social environment) in small groups - reflection on newly discovered experiences resources and strategies - mutual support regarding challenges and overcoming obstacles (based on MCII) to learn from each other in learning partnerships - conducting a mindfulness exercise together [body scan; 63] - sharing experiences with mindfulness in the group - exchange about subjective positions on the segmentation-integration continuum in the group - sharing favorite segmentation strategies in the group - exchange on how to mutually support each other in the coming weeks in learning partnerships |
| Module 4: *Staying focused – working concentrated* | - perform a 3-minute breathing exercise [12] to focus on the present and promote openness for new experiences [13] - introduce the four central processes of self-regulation: self-goal setting, self-monitoring, self-evaluation, and self-reward [25, 48, 65] - explain how to improve work organization using the selection, optimization, and compensation (SOC) model [66, 67] - set a specific goal for organizing daily work using SMART goals and MCII - learn how to use general self-regulation strategies to achieve this goal   *Daily task*: Use general self-regulation strategies to organize daily work.  *Self-regulatory element*: setting specific goals for organizing daily work and applying MCII to them; planning strategies for self-monitoring, self-evaluating, and self-rewarding when goals are met |
| Module 5: *Looking after myself – everyday rest and recharge* | - perform a 3-minute breathing exercise [12] to focus on the present and promote openness for new experiences [13] - learn about self-regulation of recovery, daily recovery in leisure time, and recovery experiences [29] in the FWD context - list all leisure activities that yield detachment, relaxation, mastery and/or control [27] - learn the importance of breaks - reflect on past recreative breaks - perform respite exercise of listening to a natural soundscape combined with a short mindfulness component, a guided imagination technique, and a savoring component [28]   *Daily task:* During work breaks, perform the savoring nature exercise to improve recovery  *Self-regulatory element*: setting specific goals and applying MCII for the daily task; planning specific ways to self-monitor, self-evaluate and self-reward progress |
| Module 6: *My strategies, my sources of strength* | - perform a 3-minute breathing exercise [12] to focus on the present and promote openness for new experiences [13] - recall the modules, reflect on insights gained from the training, judge which goals were attained, repeat and amplify the training content and make the training effects sustainable - retain and build resources perceived as relevant for reaching goals [68, 69] - using the tree metaphor, reflect on personal and contextual resources [40]   *Daily task*: In challenging situations, think about the resources needed, and reflect on a previous situation in which that resource was successfully used.  *Self-regulatory element*: identify resources through self-observation; participants reward themselves by filling in the tree with resources they identify |
| Group Session 3  (only for participants of the blended training) | - adapted version [12] of the 3-minute breathing exercise [53, 54] - short review of modules 4-6 - sharing positive experiences of the past three weeks (e.g., small successes, helpful strategies or resources within in the online training, learning partnership, or social environment) in small groups - reflection on newly discovered experiences resources and strategies - short review of the whole online training - exchange about goals for the future and feedback for each other to facilitate transfer in learning partnerships |
| General Intervention Design | *Gamification techniques [70] were included to enhance training effectiveness [71]:*   - participants viewed a system-generated illustration of a tree that added more blooms as each module was completed - blooms showed learning levels and served as a visual reward - at the beginning of each module, participants could create a personal toolbox containing their favorite exercises from the past week, to be accessed at any time   *Various sensory modalities and interactive multimedia elements* were used to enable information processing and facilitate learning:   - exercises were a mix of written explanations, audios explaining exercises, and videos of a trainer welcoming participants and explaining training content [72] - exercises were interactive and required reflection, writing, or guided imagination - audio exercises began with a brief mindfulness component [12, 28] - four fictitious models shared experiences and provided examples of personal applications to increase behavioral modelling, learning, and transfer [50, 72] |

*Note.* The description of the online training is cited from Althammer et al. [36], with the exception of the daily task in Module 6 that had been slightly modified for this study because participants had perceived the previously validated exercise as too abstract.
