# Supplementary material for: Comparing Web-Based and Blended Training for Coping With Challenges of Flexible Work Designs: Randomized Controlled Trial
Source: J Med Internet Res. 2023 Dec 19;25:e42510. doi: 10.2196/42510 (PMC10762610; doi:10.2196/42510)

**Multimedia Appendix 2**

*Screenshots of the Intervention*

Picture 1: Starting page of the training


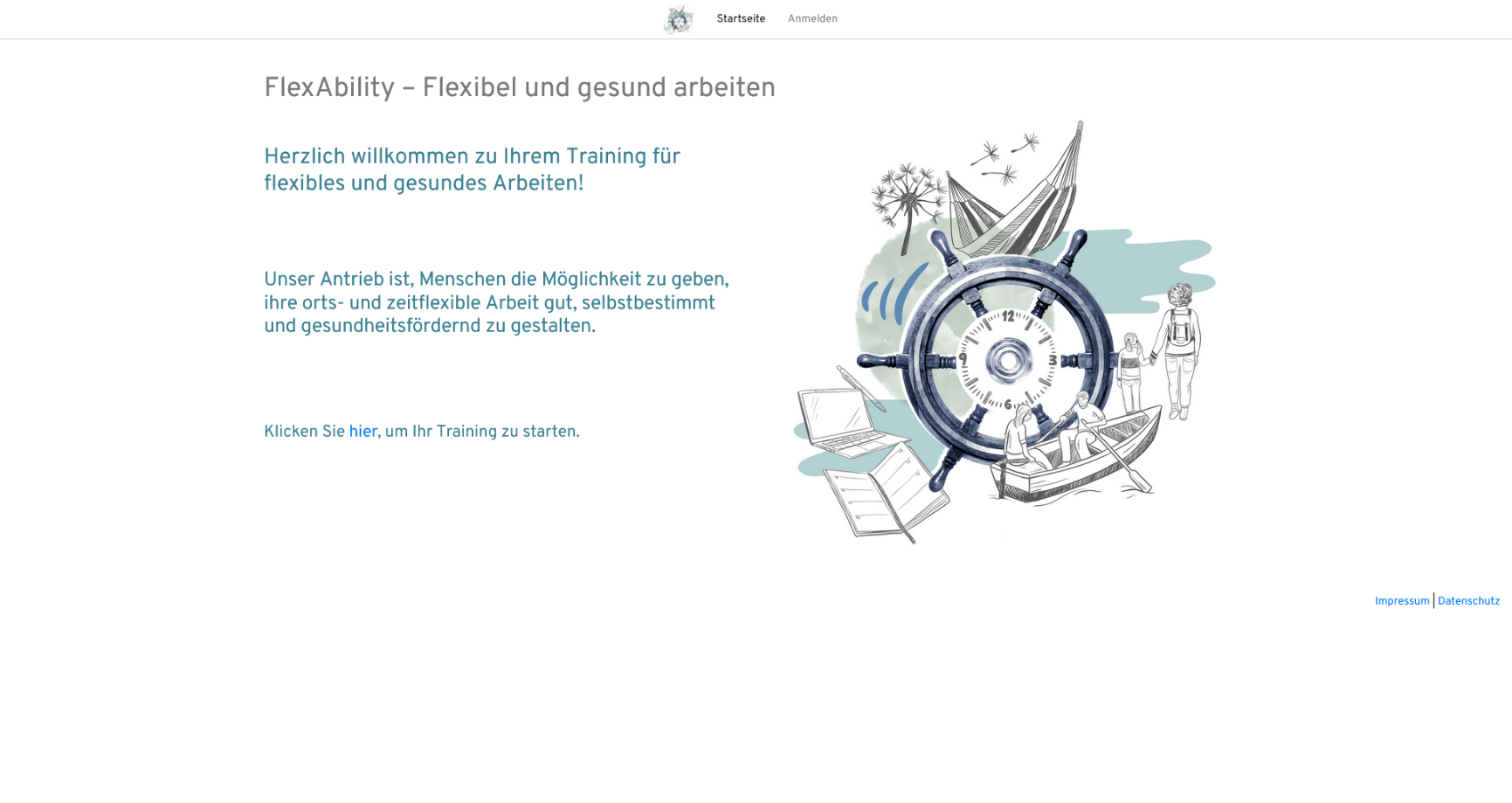


Picture 2: Starting page of Module 1 with a video of a trainer welcoming participants and explaining training content


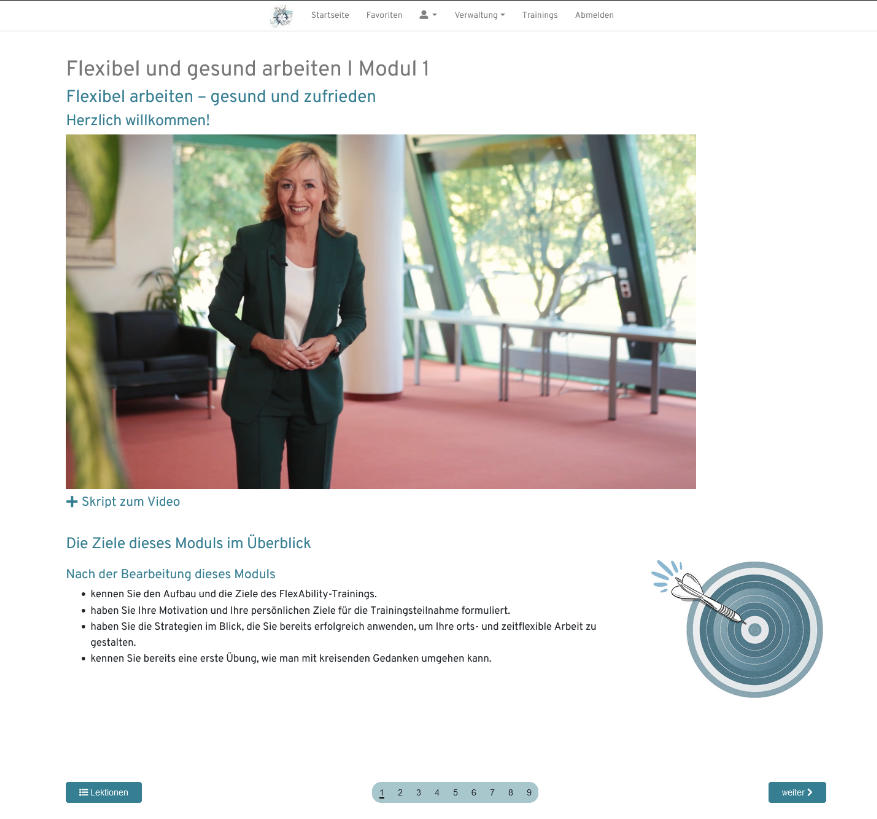


Picture 3: Examples and experiences of four fictitious models in Module 1 (extendable)


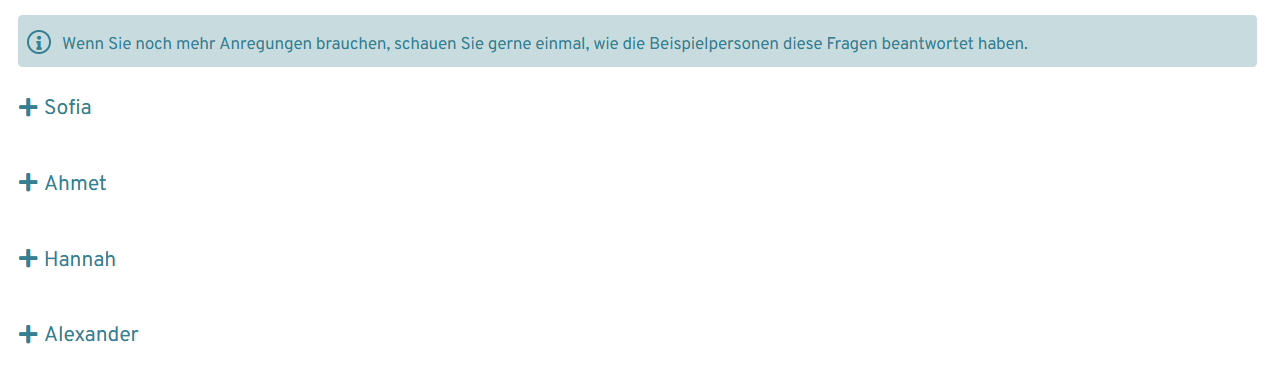


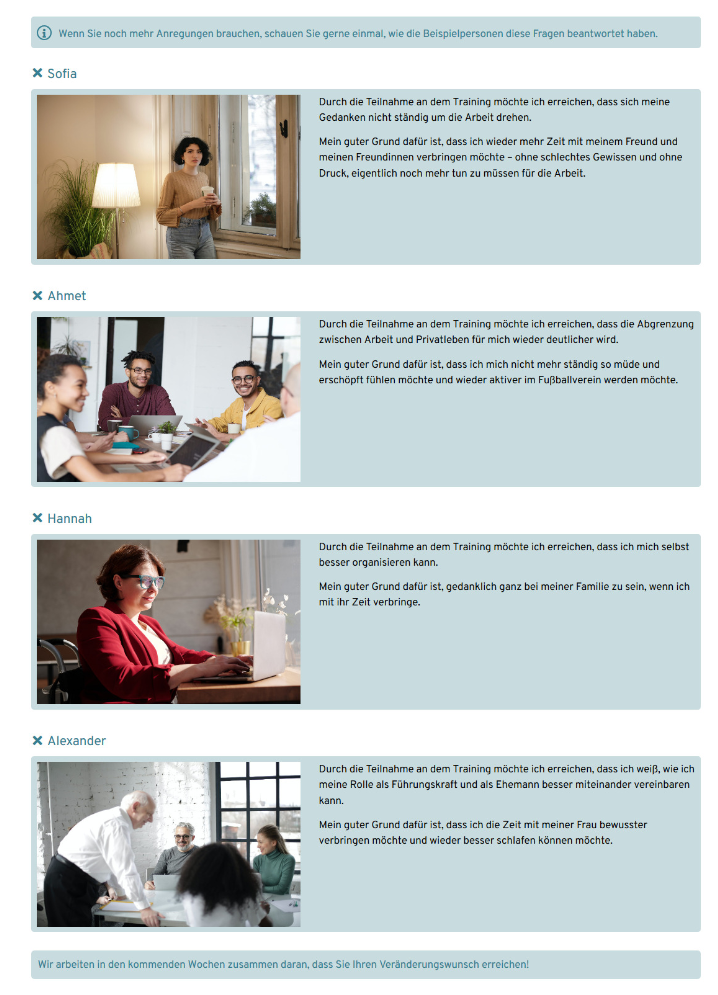


Picture 4: Self-reflection exercise in Module 2


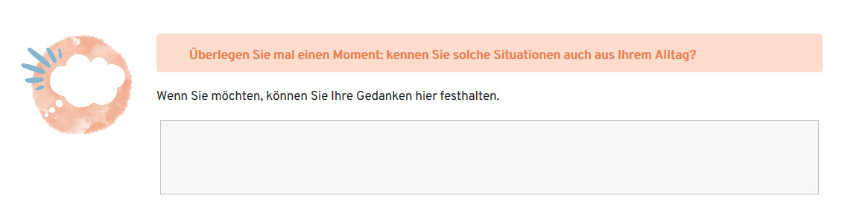


Picture 5: Audios exercise in Module 3


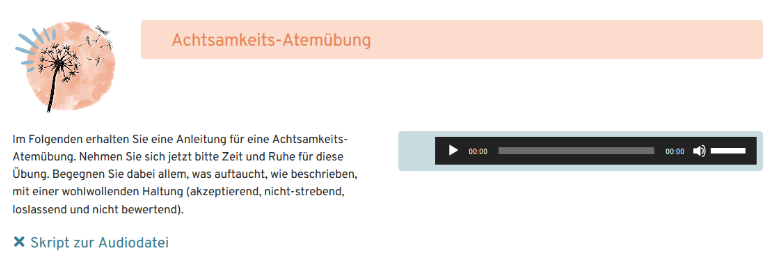


Picture 6: Written exercise in Module 4


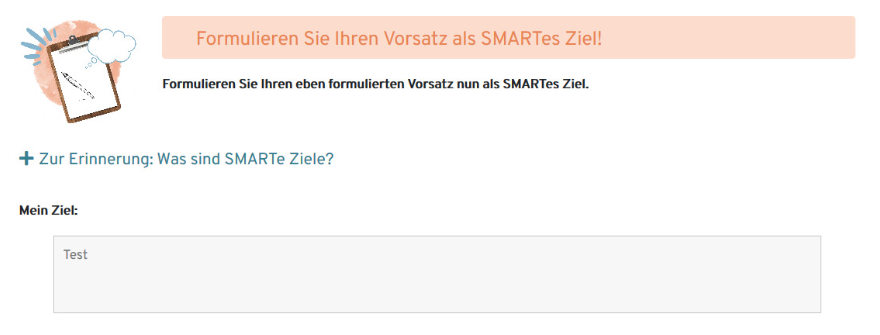


Picture 7: Personal toolbox for favorite exercises in Module 6


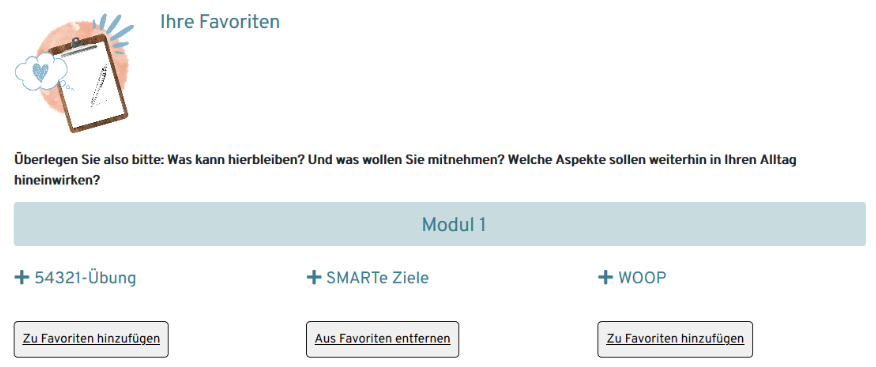


Picture 8: Illustration of the tree in Module 6


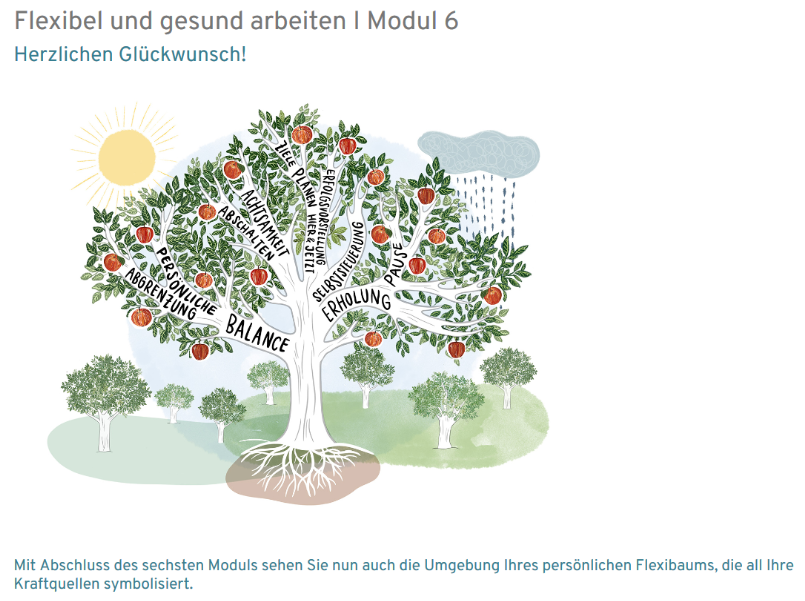

Supplement: Multimedia Appendix 2 [file jmir_v25i1e42510_app2.docx]
