# Supplementary material for: Comparing Web-Based and Blended Training for Coping With Challenges of Flexible Work Designs: Randomized Controlled Trial
Source: J Med Internet Res. 2023 Dec 19;25:e42510. doi: 10.2196/42510 (PMC10762610; doi:10.2196/42510)

**Multimedia Appendix 3**

**Table S1.** Results of the multilevel models with employment status as a moderator.

| Outcome  Predictor | *B* | *SE B* | *t* | 95% CI |
| --- | --- | --- | --- | --- |
| Psychological Detachment |  |  |  |  |
| Intercept | 3.15*** | 0.05 | 58.99 | [3.05; 3.26] |
| IG vs CG | -0.09 | 0.08 | -1.11 | [-0.24; 0.06] |
| BT vs OT | -0.02 | 0.13 | -0.14 | [-0.27; 0.23] |
| Time 1 | 0.32*** | 0.05 | 6.29 | [0.22; 0.41] |
| Time 2 | 0.29*** | 0.06 | 5.29 | [0.18; 0.40] |
| Public sector employees | 0.07 | 0.08 | 0.85 | [-0.09; 0.23] |
| Self-employed | -0.46** | 0.16 | -2.84 | [-0.77; -0.14] |
| Other employment status | 0.43 | 0.49 | 0.88 | [-0.52; 1.38] |
| IG vs CG x Time 1 | 0.19** | 0.07 | 2.83 | [0.06; 0.33] |
| IG vs CG x Time 2 | 0.26*** | 0.08 | 3.42 | [0.11; 0.40] |
| BT vs OT x Time 1 | 0.16 | 0.13 | 1.27 | [-0.08; 0.41] |
| BT vs OT x Time 2 | 0.14 | 0.14 | 0.98 | [-0.13; 0.41] |
| Public sector employees x Time 1 | -0.15 | 0.08 | -1.92 | [-0.30; 0.00] |
| Public sector employees x Time 2 | -0.14 | 0.08 | -1.62 | [-0.30; 0.03] |
| Self-employed x Time 1 | -0.11 | 0.15 | -0.73 | [-0.41; 0.18] |
| Self-employed x Time 2 | -0.18 | 0.17 | -1.09 | [-0.51; 0.14] |
| Other employment status x Time 1 | -0.01 | 0.43 | -0.03 | [-0.84; 0.81] |
| Other employment status x Time 2 | -0.07 | 0.45 | -0.16 | [-0.95; 0.81] |
| Public sector employees x IG vs CG | 0.00 | 0.12 | 0.03 | [-0.22; 0.23] |
| Public sector employees x BT vs OT | 0.02 | 0.20 | 0.10 | [-0.36; 0.40] |
| Self-employed x IG vs CG | 0.28 | 0.24 | 1.19 | [-0.18; 0.75] |
| Self-employed x BT vs OT | -0.12 | 0.37 | -0.33 | [-0.84; 0.60] |
| Other employment status x IG vs CG | -0.83 | 0.72 | -1.15 | [-2.24; 0.58] |
| Other employment status x BT vs OT | 0.77 | 1.14 | 0.68 | [-1.44; 2.97] |
| IG vs CG x Time 1 x Public sector employees | -0.08 | 0.10 | -0.79 | [-0.28; 0.12] |
| IG vs CG x Time 2 x Public sector employees | -0.16 | 0.12 | -1.41 | [-0.39; 0.06] |
| BT vs OT x Time 1 x Public sector employees | -0.10 | 0.20 | -0.49 | [-0.47; 0.28] |
| BT vs OT x Time 2 x Public sector employees | 0.01 | 0.21 | 0.07 | [-0.40; 0.43] |
| IG vs CG x Time 1 x Self-employed | -0.12 | 0.22 | -0.53 | [-0.55; 0.32] |
| IG vs CG x Time 2 x Self-employed | -0.20 | 0.26 | -0.75 | [-0.70; 0.31] |
| BT vs OT x Time 1 x Self-employed | 0.19 | 0.36 | 0.53 | [-0.50; 0.89] |
| BT vs OT x Time 2 x Self-employed | -0.12 | 0.38 | -0.32 | [-0.85; 0.61] |
| IG vs CG x Time 1 x Other employment status | -0.14 | 0.61 | -0.23 | [-1.31; 1.03] |
| IG vs CG x Time 2 x Other employment status | -0.04 | 0.65 | -0.06 | [-1.29; 1.21] |
| BT vs OT x Time 1 x Other employment status | -1.50 | 1.04 | -1.44 | [-3.50; 0.51] |
| BT vs OT x Time 2 x Other employment status | -0.98 | 1.10 | -0.89 | [-3.12; 1.16] |
| Satisfaction with Work-Life Balance |  |  |  |  |
| Intercept | 3.33*** | 0.05 | 63.48 | [3.23; 3.43] |
| IG vs CG | 0.00 | 0.08 | 0.01 | [-0.15; 0.15] |
| BT vs OT | -0.25 | 0.13 | -2.00 | [-0.49; -0.01] |
| Time 1 | 0.12* | 0.05 | 2.53 | [0.03; 0.21] |
| Time 2 | 0.19*** | 0.05 | 3.51 | [0.08; 0.29] |
| Public sector employees | -0.01 | 0.08 | -0.11 | [-0.16; 0.15] |
| Self-employed | -0.28 | 0.16 | -1.75 | [-0.58; 0.03] |
| Other employment status | 0.34 | 0.48 | 0.70 | [-0.59; 1.27] |
| IG vs CG x Time 1 | 0.21*** | 0.06 | 3.31 | [0.09; 0.33] |
| IG vs CG x Time 2 | 0.21** | 0.07 | 2.82 | [0.06; 0.35] |
| BT vs OT x Time 1 | 0.26* | 0.12 | 2.24 | [0.04; 0.49] |
| BT vs OT x Time 2 | 0.12 | 0.14 | 0.87 | [-0.14; 0.38] |
| Public sector employees x Time 1 | -0.07 | 0.07 | -0.94 | [-0.20; 0.07] |
| Public sector employees x Time 2 | -0.04 | 0.08 | -0.47 | [-0.20; 0.12] |
| Self-employed x Time 1 | 0.01 | 0.14 | 0.10 | [-0.26; 0.29] |
| Self-employed x Time 2 | 0.05 | 0.17 | 0.30 | [-0.27; 0.37] |
| Other employment status x Time 1 | -0.84* | 0.39 | -2.13 | [-1.60; -0.08] |
| Other employment status x Time 2 | -0.55 | 0.45 | -1.24 | [-1.42; 0.31] |
| Public sector employees x IG vs CG | -0.01 | 0.11 | -0.07 | [-0.23; 0.21] |
| Public sector employees x BT vs OT | 0.35 | 0.19 | 1.83 | [-0.02; 0.73] |
| Self-employed x IG vs CG | 0.18 | 0.23 | 0.76 | [-0.28; 0.63] |
| Self-employed x BT vs OT | 0.13 | 0.36 | 0.35 | [-0.58; 0.83] |
| Other employment status x IG vs CG | -0.83 | 0.71 | -1.17 | [-2.21; 0.55] |
| Other employment status x BT vs OT | -0.25 | 1.11 | -0.22 | [-2.41; 1.91] |
| IG vs CG x Time 1 x Public sector employees | -0.19* | 0.10 | -2.02 | [-0.38; -0.01] |
| IG vs CG x Time 2 x Public sector employees | -0.08 | 0.11 | -0.70 | [-0.30; 0.14] |
| BT vs OT x Time 1 x Public sector employees | -0.23 | 0.18 | -1.29 | [-0.58; 0.12] |
| BT vs OT x Time 2 x Public sector employees | -0.12 | 0.21 | -0.59 | [-0.53; 0.28] |
| IG vs CG x Time 1 x Self-employed | 0.11 | 0.21 | 0.51 | [-0.29; 0.51] |
| IG vs CG x Time 2 x Self-employed | 0.15 | 0.25 | 0.60 | [-0.34; 0.64] |
| BT vs OT x Time 1 x Self-employed | 0.02 | 0.33 | 0.06 | [-0.62; 0.66] |
| BT vs OT x Time 2 x Self-employed | 0.05 | 0.37 | 0.14 | [-0.66; 0.76] |
| IG vs CG x Time 1 x Other employment status | -0.18 | 0.56 | -0.32 | [-1.27; 0.90] |
| IG vs CG x Time 2 x Other employment status | 0.18 | 0.64 | 0.28 | [-1.05; 1.42] |
| BT vs OT x Time 1 x Other employment status | -2.18* | 0.96 | -2.27 | [-4.03; -0.33] |
| BT vs OT x Time 2 x Other employment status | -1.46 | 1.08 | -1.34 | [-3.55; 0.65] |
| Well-being |  |  |  |  |
| Intercept | 3.49*** | 0.06 | 58.13 | [3.37; 3.60] |
| IG vs CG | 0.03 | 0.09 | 0.38 | [-0.14; 0.20] |
| BT vs OT | -0.21 | 0.14 | -1.46 | [-0.49; 0.07] |
| Time 1 | 0.18*** | 0.05 | 3.30 | [0.07; 0.28] |
| Time 2 | 0.17** | 0.06 | 2.65 | [0.04; 0.29] |
| Public sector employees | -0.02 | 0.09 | -0.19 | [-0.19; 0.16] |
| Self-employed | -0.08 | 0.18 | -0.47 | [-0.43; 0.27] |
| Other employment status | 0.88 | 0.55 | 1.60 | [-0.19; 1.94] |
| IG vs CG x Time 1 | 0.20** | 0.07 | 2.70 | [0.06; 0.34] |
| IG vs CG x Time 2 | 0.27** | 0.09 | 3.15 | [0.10; 0.44] |
| BT vs OT x Time 1 | 0.10 | 0.14 | 0.76 | [-0.16; 0.37] |
| BT vs OT x Time 2 | 0.18 | 0.16 | 1.11 | [-0.13; 0.48] |
| Public sector employees x Time 1 | -0.09 | 0.08 | -1.12 | [-0.25; 0.07] |
| Public sector employees x Time 2 | 0.01 | 0.10 | 0.06 | [-0.18; 0.19] |
| Self-employed x Time 1 | 0.05 | 0.17 | 0.33 | [-0.27; 0.37] |
| Self-employed x Time 2 | -0.03 | 0.19 | -0.18 | [-0.41; 0.34] |
| Other employment status x Time 1 | -1.13* | 0.46 | -2.46 | [-2.02; -0.24] |
| Other employment status x Time 2 | -1.11* | 0.52 | -2.14 | [-2.11; -0.10] |
| Public sector employees x IG vs CG | 0.03 | 0.13 | 0.19 | [-0.23; 0.28] |
| Public sector employees x BT vs OT | 0.40 | 0.22 | 1.81 | [-0.03; 0.83] |
| Self-employed x IG vs CG | 0.23 | 0.27 | 0.86 | [-0.29; 0.75] |
| Self-employed x BT vs OT | -0.13 | 0.42 | -0.31 | [-0.94; 0.68] |
| Other employment status x IG vs CG | -0.27 | 0.81 | -0.33 | [-1.85; 1.31] |
| Other employment status x BT vs OT | -0.09 | 1.27 | -0.07 | [-2.57; 2.38] |
| IG vs CG x Time 1 x Public sector employees | -0.21 | 0.11 | -1.83 | [-0.42; 0.01] |
| IG vs CG x Time 2 x Public sector employees | -0.19 | 0.13 | -1.45 | [-0.44; 0.06] |
| BT vs OT x Time 1 x Public sector employees | -0.13 | 0.21 | -0.62 | [-0.54; 0.28] |
| BT vs OT x Time 2 x Public sector employees | 0.04 | 0.24 | 0.17 | [-0.43; 0.51] |
| IG vs CG x Time 1 x Self-employed | 0.09 | 0.24 | 0.39 | [-0.38; 0.56] |
| IG vs CG x Time 2 x Self-employed | -0.18 | 0.30 | -0.61 | [-0.75; 0.39] |
| BT vs OT x Time 1 x Self-employed | 0.41 | 0.39 | 1.05 | [-0.34; 1.16] |
| BT vs OT x Time 2 x Self-employed | -0.22 | 0.43 | -0.52 | [-1.05; 0.61] |
| IG vs CG x Time 1 x Other employment status | -0.35 | 0.66 | -0.54 | [-1.62; 0.91] |
| IG vs CG x Time 2 x Other employment status | -1.01 | 0.74 | -1.38 | [-2.44; 0.41] |
| BT vs OT x Time 1 x Other employment status | -1.76 | 1.12 | -1.57 | [-3.93; 0.40] |
| BT vs OT x Time 2 x Other employment status | -3.20* | 1.26 | -2.54 | [-5.64; -0.76] |

*Note. N* = 1132. IG vs CG = Intervention Groups vs. Control Group; BT vs OT = Blended Training vs. Online Training. * indicates *p* < .05. ** indicates *p* < .01. *** indicates *p* < .001. Zero is not included in the reported confidence intervals if the lower and upper bound of the confidence interval have the same sign. In these reported confidence intervals, numbers not equal to zero would appear if more decimal places were reported.

**Figure S1**

Means of outcome variables for the intervention groups and control group at baseline (T0), postintervention time point (T1), and 4-week follow-up (T2) stratified for employment status.


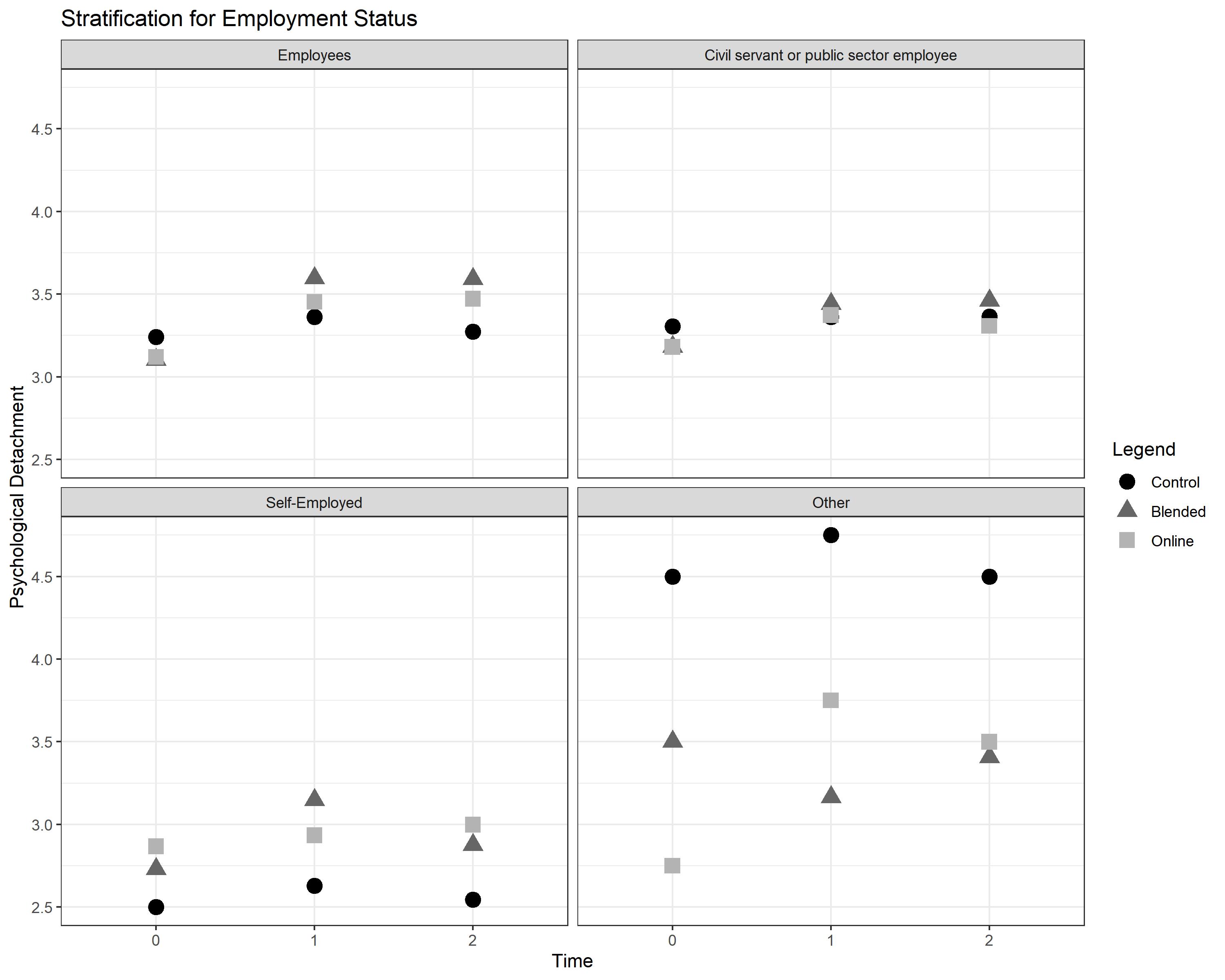

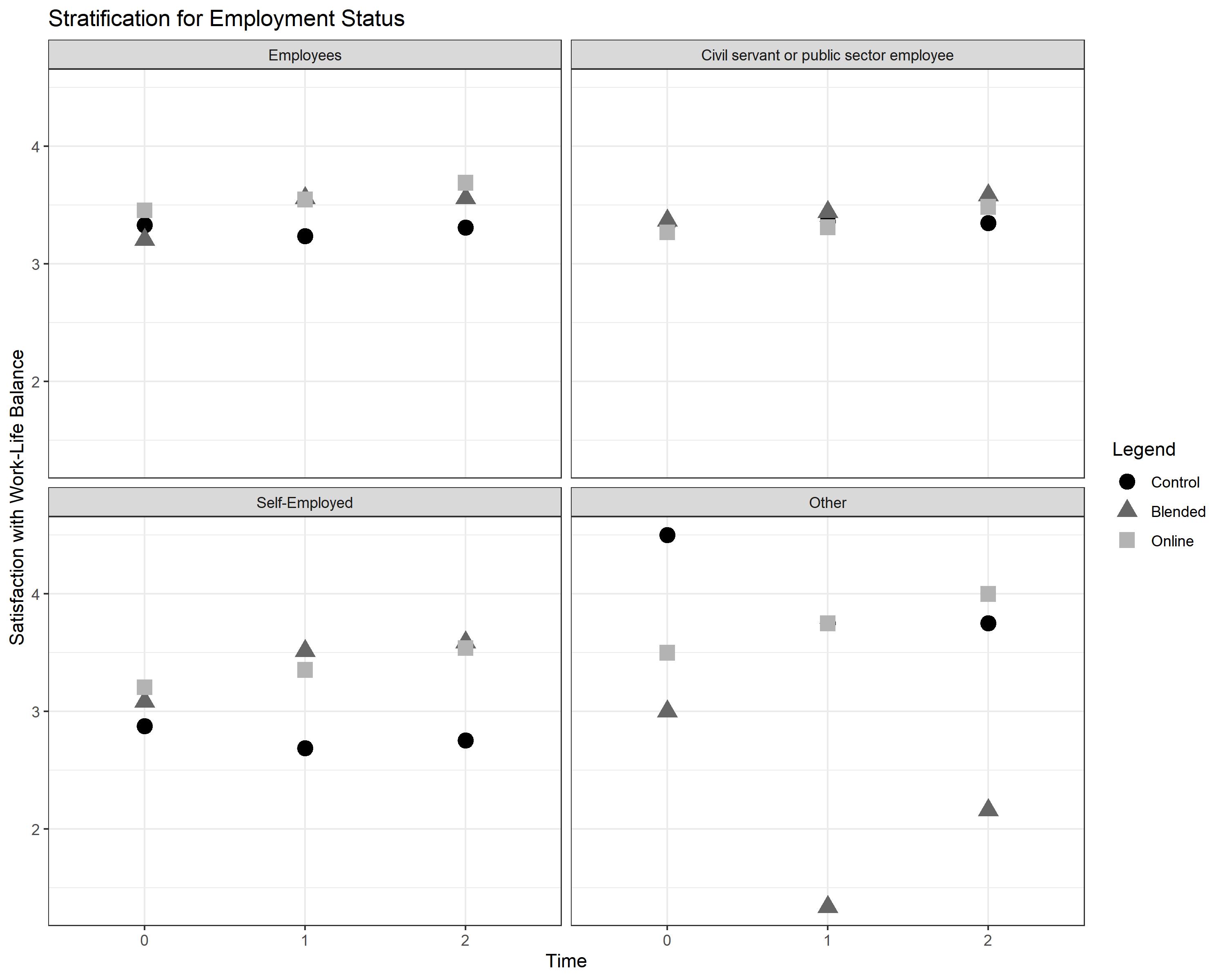

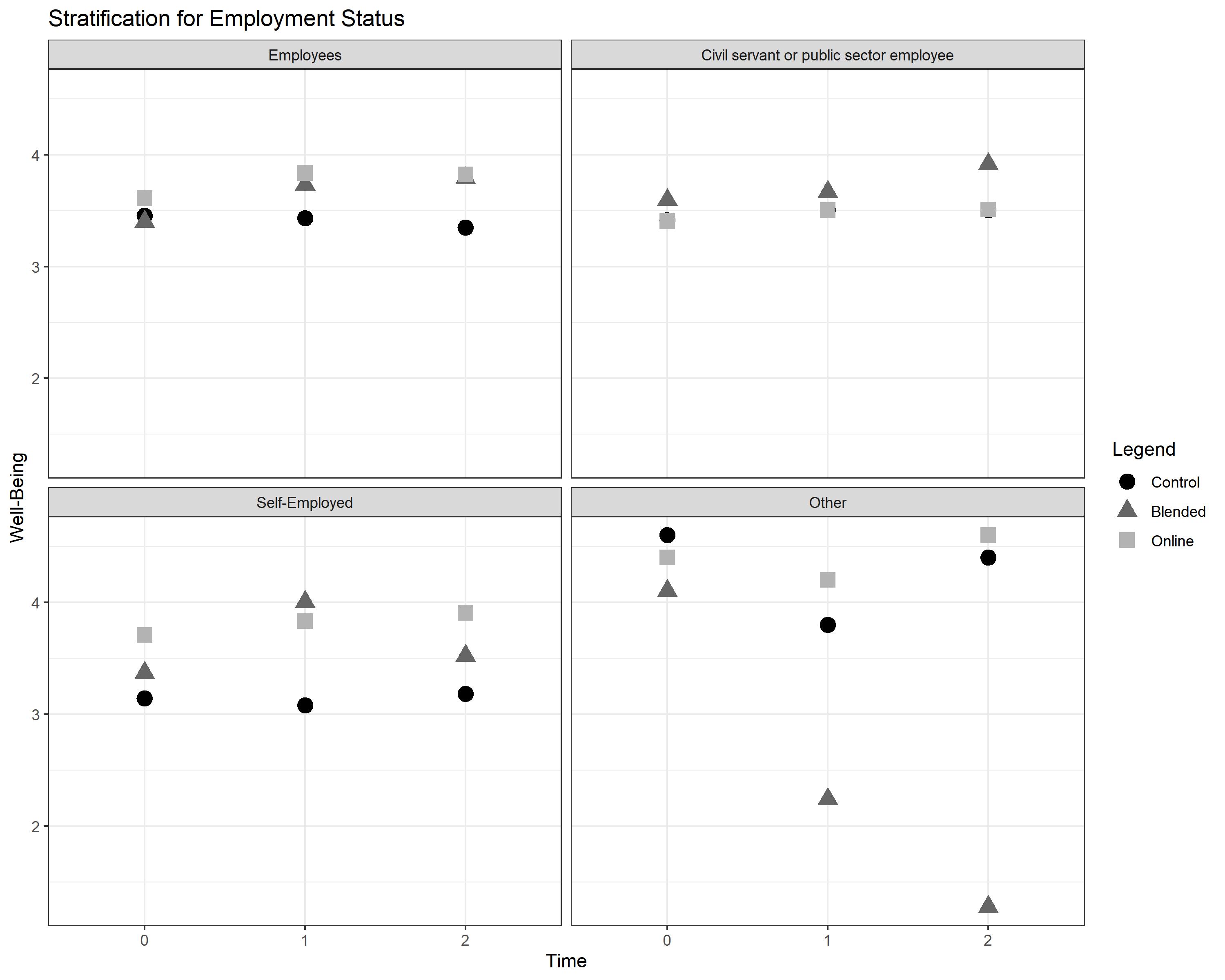

Supplement: Multimedia Appendix 3 [file jmir_v25i1e42510_app3.docx]
